# Hebbian Wiring Plasticity Generates Efficient Network Structures for Robust Inference with Synaptic Weight Plasticity

**DOI:** 10.3389/fncir.2016.00041

**Published:** 2016-05-31

**Authors:** Naoki Hiratani, Tomoki Fukai

**Affiliations:** ^1^Department of Complexity Science and Engineering, The University of TokyoKashiwa, Japan; ^2^Laboratory for Neural Circuit Theory, RIKEN Brain Science InstituteWako, Japan

**Keywords:** synaptic plasticity, synaptogenesis, neural decoding, computational model, connectomics

## Abstract

In the adult mammalian cortex, a small fraction of spines are created and eliminated every day, and the resultant synaptic connection structure is highly nonrandom, even in local circuits. However, it remains unknown whether a particular synaptic connection structure is functionally advantageous in local circuits, and why creation and elimination of synaptic connections is necessary in addition to rich synaptic weight plasticity. To answer these questions, we studied an inference task model through theoretical and numerical analyses. We demonstrate that a robustly beneficial network structure naturally emerges by combining Hebbian-type synaptic weight plasticity and wiring plasticity. Especially in a sparsely connected network, wiring plasticity achieves reliable computation by enabling efficient information transmission. Furthermore, the proposed rule reproduces experimental observed correlation between spine dynamics and task performance.

## Introduction

The amplitude of excitatory and inhibitory postsynaptic potentials (EPSPs and IPSPs), often referred to as synaptic weight, is considered a fundamental variable in neural computation (Bliss and Collingridge, 1993; Dayan and Abbott, 2005). In the mammalian cortex, excitatory synapses often show large variations in EPSP amplitudes (Song et al., 2005; Ikegaya et al., 2013; Buzsáki and Mizuseki, 2014), and the amplitude of a synapse can be stable over trials (Lefort et al., 2009) and time (Yasumatsu et al., 2008), enabling rich information capacity compared with that at binary synapses (Brunel et al., 2004; Hiratani et al., 2013). In addition, synaptic weight shows a wide variety of plasticity which depend primarily on the activity of presynaptic and postsynaptic neurons (Caporale and Dan, 2008; Feldman, 2009). Correspondingly, previous theoretical results suggest that under appropriate synaptic plasticity, a randomly connected network is computationally sufficient for various tasks (Maass et al., 2002; Ganguli and Sompolinsky, 2012).

On the other hand, it is also known that synaptic wiring plasticity and the resultant synaptic connection structure are crucial for computation in the brain (Chklovskii et al., 2004; Holtmaat and Svoboda, 2009). Elimination and creation of dendritic spines are active even in the brain of adult mammalians. In rodents, the spine turnover rate is up to 15% per day in sensory cortex (Holtmaat et al., 2005) and 5% per day in motor cortex (Zuo et al., 2005). Recent studies further revealed that spine dynamics are tightly correlated with the performance of motor-related tasks (Xu et al., 2009; Yang et al., 2009). Previous modeling studies suggest that wiring plasticity helps memory storage (Poirazi and Mel, 2001; Stepanyants et al., 2002; Knoblauch et al., 2010). However, in those studies, EPSP amplitude was often assumed to be a binary variable, and wiring plasticity was performed in a heuristic manner. Thus, it remains unknown what should be encoded by synaptic connection structure when synaptic weights have a rich capacity for representation, and how such a connection structure can be achieved through a local spine elimination and creation mechanism, which is arguably noisy and stochastic (Kasai et al., 2010).

To answer these questions, we constructed a theoretical model of an inference task. We first studied how sparse connectivity affects the performance of the network by analytic consideration and information theoretic evaluations. Then, we investigated how synaptic weights and connectivity should be organized to perform robust inference, especially under the presence of variability in the input structure. Based on these insights, we proposed a local unsupervised rule for wiring and synaptic weight plasticity. In addition, we demonstrated that connection structure and synaptic weight learn different components under a dynamic environment, enabling robust computation. Lastly, we investigated whether the model is consistent with various experimental results on spine dynamics.

## Results

### Connection structure reduces signal variability in sparsely connected networks

**Figure 1:**
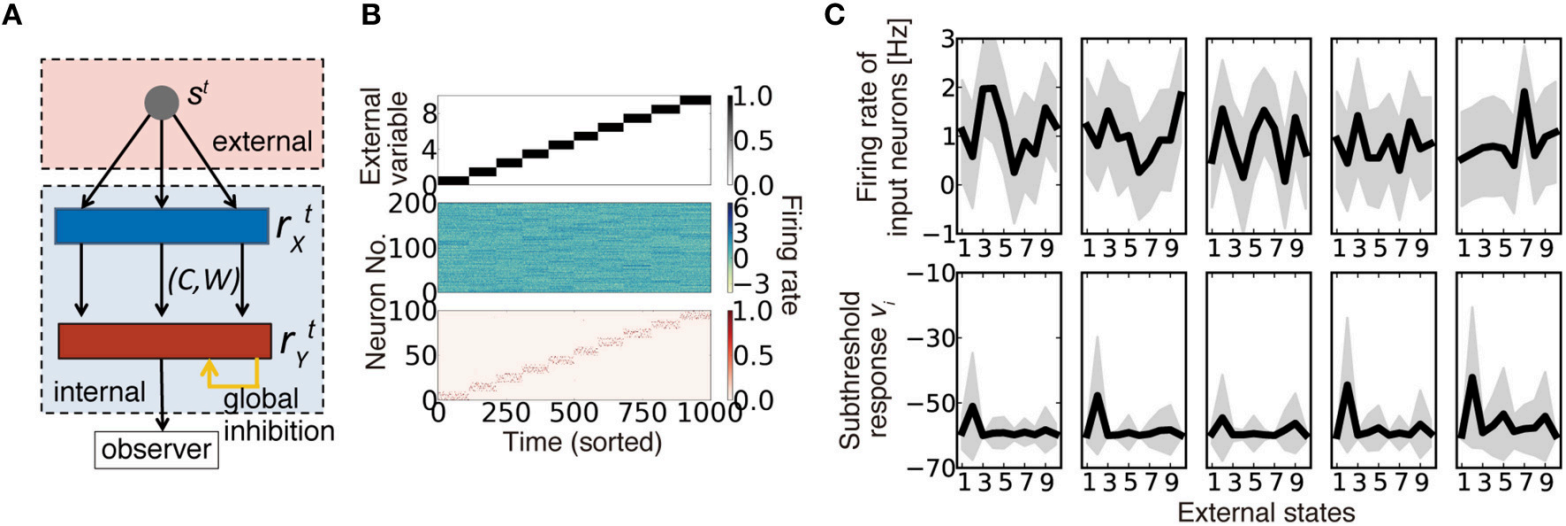
**Description of the model. (A)** Schematic diagram of the model. **(B)** An example of model behavior calculated at ρ = 0.16, when the synaptic connection is organized using the weight-coding scheme. The top panel represents the external variable, which takes an integer 0 to 9 in the simulation. The middle panel is the response of input neurons, and the bottom panel shows the activity of output neurons. In the simulation, each external state was randomly presented, but here the trials are sorted in ascending order. **(C)** Examples of neural activity in a simulation. Graphs on the top row represent the average firing rates of five randomly sampled input neurons for given external states (black lines) and their standard deviation (gray shadows). The bottom graphs are subthreshold responses of output neurons that represent the external state *s* = 1. Because the boundary condition for the membrane parameter $v_{i}\equiv \underset{j}{\sum }c_{ij}\left(w_{ij}r_{X,j}^{t}-h_{w}\right)$ was introduced as $v_{i}>{\text{max}}_{i^{\prime }}\left\{v_{i^{\prime }}-v_{d}\right\}$, *v*_*i*_ is typically bounded at −*v*_*d*_. Note that *v*_*i*_ is the unnormalized log-likelihood, and the units on the y-axis are arbitrary.

What should be represented by synaptic connections and their weights, and how are those representations acquired? To explore the answers to these questions, we studied a hidden variable estimation task (Figure 1A), which appears in various stages of neural information processing (Beck et al., 2008; Lochmann and Deneve, 2011). In the task, at every time *t*, one hidden state is sampled with equal probability from *p* number of external states *s*^*t*^ = {*0,1,…,p* − *1*}. Neurons in the input layer show independent stochastic responses $r_{X,j}^{t}$ ~ *N*(θ_*jμ*_, σ_*X*_) due to various noises (Figure 1B, middle), where $r_{X,j}^{t}$ is the firing rate of input neuron *j* at time *t*, θ_*jμ*_ is the average firing rate of neuron *j* to the stimulus μ, and σ_*X*_ is the constant noise amplitude (see Table 1 for the definitions of variables and parameters). Although, we used Gaussian noise for analytical purposes, the following argument is applicable for any stochastic response that follows a general exponential family, including Poisson firing (Supplementary Figure 1). Neurons in the output layer estimate the hidden variable from input neuron activity and represent the variable with population firing {$r_{Y,i}^{t}$}, where *i* = 1,2,…*N* are indices of output neurons. This task is computationally difficult because most input neurons have mixed selectivity for several hidden inputs, and the responses of the input neurons are highly stochastic (Figure 1C). Let us assume that the dynamics of output neurons are written as follows:

(1) $$\begin{array}{l} r_{Y,i}^{\;t}=r_{Y}^{o}\exp\left[\sum_{j\;=\;1}^{M}c_{ij}\left(w_{ij}r_{X,j}^{t}-h_{w}\right)-I_{inh}^{\;t}\right],\\ I_{inh}^{\;t}=\log\left[\sum_{i=1}^{N}\exp\left(\sum_{j\;=\;1}^{M}c_{ij}\left[w_{ij}r_{X,j}^{t}-h_{w}\right]\right)\right], \end{array}$$

where *c*_*ij*_ (= 0 or 1) represents connectivity from input neuron *j* to output neuron *i, w*_*ij*_ is its synaptic weight (EPSP size), and *h*_*w*_ is the threshold. *M* and *N* are population sizes of the input and output layers, respectively. In the model, all feedforward connections are excitatory, and the inhibitory input is provided as the global inhibition $I_{inh}^{t}$.

**Table 1:** **Definitions of main variables and parameters**.

**Name**	**Description**	**Definition**
*s^*t*^*	Hidden external state at time *t*	Section Details of Simulation
$r_{X,j}^{t}$	Firing rate of input neuron *j* at time *t*	Equation (5)
$r_{Y,i}^{t}$	Firing rate of output neuron *i* at time *t*	Equation (1)
*w_*ij*_*	Synaptic weight from input neuron *i* to output neuron *j*	Constant (Figures 1–3) Equation (2) (Figures 4–8)
*c_*ij*_*	Number of connection from input neuron *i* to output neuron *j* (Note that here *c_*ij*_* = 0 or 1)	Section Synaptic Connection Learning
ρ_*ij*_	Connection probability from input neuron *i* to output neuron *j*	Constant (Figures 1–4) Equation (3) (Figures 5, 6) Equation (4) (Figures 6I, 7, 8)
	The dual Hebbian rule	Equation (2) + Equation (3)
	The approximated dual Hebbian rule	Equation (2) + Equation (4)
*θ_*jμ*_*	Response parameter of neuron *j* to hidden state μ	Section Gaussian Model, Poisson Model
*q_*jμ*_*	Normalized response parameter of neuron *j* to hidden state μ. Especially in the Gaussian model, *q_*jμ*_* = *θ_*jμ*_*/$\sigma_{X}^{2}$	*q_*jμ*_* = *h*(*θ_*jμ*_*)
*Ω_μ_*	Set of output neurons that selective for hidden state μ	Section Accuracy of Estimation
*h_*w*_*	Input threshold	Section Details of Simulation
*σ_*X*_*	Noise in input neuron firing rate	*σ_*X*_* = 1.0
γ	Parameter for sparseness of connectivity	Sections Weight Coding and Connectivity Coding and Dual Coding and Cut-Off Coding
*b_*h*_*	Strength of homeostatic plasticity	Equation (2)
*τ_*c*_*	Timescale of rewiring	Section Synaptic Connection Learning
κ_*m*_	Ratio between constant and variable component in *θ_*jμ*_*	θ_*jμ*_ = $\frac{1}{Z}\left[\kappa_{m}\theta_{j\mu }^{const}+\left(1-\kappa_{m}\right)\theta_{j\mu }^{\text{var}}\right]$
*θ_*const*_, θ_*var*_*	Two component of input structures used in Figure 6	Section Gaussian Model
*T_2_*	Interval between update of the variable component θ_*var*_	*T*_2_ = 10^5^
*θ_*ctrl*,_θ_*training*_*	Two input structures used for modeling control and training phases in Figure 8	Section Gaussian Model

**Figure 2:**
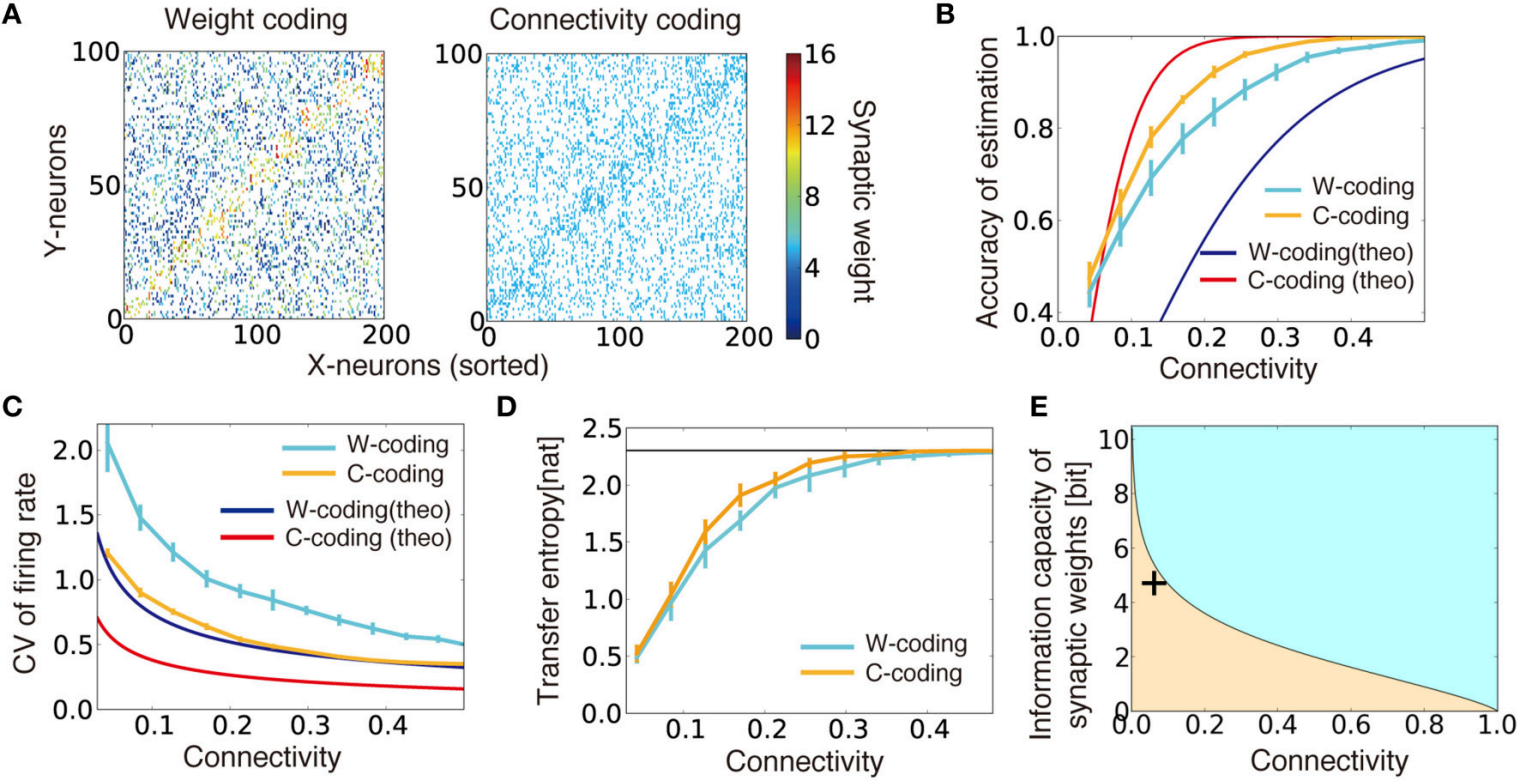
**Performance comparison between connectivity coding and weight coding. (A)** Examples of synaptic weight matrices in weight coding (W-coding) and connectivity coding (C-coding) schemes calculated at γ = 0.2. X-neurons were sorted by their selectivity for external states, and colors represent synaptic weights. **(B)** Comparison of the performance between connectivity coding and weight coding schemes at various sparseness of connectivity. Orange and cyan lines are simulation results. The error bars represent standard deviation over 10 independent simulations. In the following panels, error bars are trial variability over 10 simulations. Red and blue lines are analytical results. **(C)** Analytically evaluated coefficient of variation (CV) of output firing rate and corresponding simulation results. For simulation results, the variance was evaluated over whole output neurons from their firing rates for their selective external states. **(D)** Estimated maximum transfer entropy for two coding strategies. Black horizontal line is the maximal information log_*e*_*p*. **(E)** Relative information capacity of connection structure vs. synaptic weight is shown at various values of synaptic connectivity. In the orange (cyan) area, the synaptic connectivity has higher (lower) information capacity than the synaptic weights. Plus symbol represents the data point obtained from CA3-to-CA1 connections.

If the feedforward connection is all-to-all (i.e., *c*_*ij*_ = 1 for all *i*,*j* pairs), by setting the weights as $w_{ij}=q_{j\mu }\equiv \theta_{j\mu }∕\sigma_{X}^{2}$ for output neuron *i* that represents external state μ, the network gives an optimal inference from the given firing rate vector $r_{X}^{t}$, because the value *q*_*jμ*_ represents how much evidence the firing rate of neuron *j* provides for a particular external state μ. (For details, see Sections Model dynamics and Gaussian Model). However, if the connectivity between the two layers is sparse, as in most regions of the brain (Potjans and Diesmann, 2014), optimal inference is generally unattainable because each output neuron can obtain a limited set of information from the input layer. How should one choose connection structure and synaptic weights in such a case? Intuitively, we could expect that if we randomly eliminate connections while keeping the synaptic weights of output neuron *i* that represents external state μ as *w*_*ij*_ ∝ *q*_*jμ*_ (below, we call it as weight coding), the network still works at a near-optimal accuracy. On the other hand, even if the synaptic weight is a constant value, if the connection probability is kept at ρ_*ij*_∝*q*_*jμ*_ (i.e., connectivity coding; see Section Weight Coding and Connectivity Coding for details of coding strategies), the network is expected to achieve near-optimal performance. Figure 2A describes the connection matrices between input/output layers in two strategies. In the weight coding, if we sort input neurons with their preferred external states, the diagonal components of the connection matrix show high synaptic weights, whereas in the connectivity coding, the diagonal components show dense connection (Figure 2A). Both of realizations asymptotically converge to optimal solution when the number of neurons in the middle layer is sufficiently large, though in a finite network, not strictly optimal under given constraints. In addition, both of them are obtainable through biologically plausible local Hebbian learning rules as we demonstrate in subsequent sections.

We evaluated the accuracy of the external state estimation using a bootstrap method (Section Accuracy of Estimation) for both coding strategies. Under intermediate connectivity, both strategies showed reasonably good performance (as in Figure 1B, bottom). Intriguingly, in sparsely connected networks, the connectivity coding outperformed the weight coding, despite its binary representation (Figure 2B, cyan/orange lines). The analytical results confirmed this tendency (Figure 2B, red/blue lines; see Section Evaluation of Performances in Weight Coding and Connectivity Coding for the Details) and indicated that the firing rates of output neurons selective for the given external state show less variability in connectivity coding than in the weight coding, enabling more reliable information transmission (Figure 2C). To further understand this phenomenon, we evaluated the maximum transfer entropy of the feed forward connections: $T_{E}={\langle H\left(s^{t}\right)-H\left(s^{t}|r_{X}^{t},C\right)\rangle }_{t}$. Because of limited connectivity, each output neuron obtains information only from the connected input neurons. Thus, the transfer entropy was typically lower under sparse than under dense connections in both strategies (Figure 2D). However, in the connectivity coding scheme, because each output neuron can get information from relevant input neurons, the transfer entropy became relatively large compared to the weight coding (orange line in Figure 2D). Therefore, analyses from both statistical and information theory-based perspectives confirm the advantage of connectivity coding over the weight coding in the sparse regions.

The result above can also be extended to arbitrary feedforward networks as below. For a feedforward network of *M* input and *N* output neurons with connection probability ρ, information capacity of connections is given as *I*_*C*_ (ρ) ≡ log_*MN*_ *C*_ρ*MN*_ ≈ *MN* · *H*(ρ), where *H* represents the entropy function *H*(ρ) ≡ −ρlogρ − (1 − ρ) log (1 − ρ). Similarly, for a given connections between two layers, information capacity of synaptic weights is written as *I*_*w*_(ρ) ≡ ρ*MN* log *b*, where *b* is the number of distinctive synaptic states (Varshney et al., 2006). Therefore, when the connection probability ρ satisfies *b* = exp [*H*(ρ) ∕ ρ], synaptic connections and weights have the same information capacities. This means that, as depicted in Figure 2E, in a sparsely connected network, synaptic connections tend to have larger relative information capacity, compared to a dense network with the same *b*. This result is consistent with the model above, because stochastic firing of presynaptic neuron can be regarded as synaptic noise even though synaptic weights have an infinitely high resolution in the model. Furthermore, in the CA3-to-CA1 connection of mice, connection probability is estimated to be around 6% (Sayer et al., 1990), and information capacity of synaptic weight is around 4.7 bits (Bartol et al., 2015), thus the connection structure should also play an active role in neural coding in the real brain (data point in Figure 2E).

### Dual coding by synaptic weights and connections enables robust inference

In the section above, we demonstrated that a random connection structure highly degrades information transmission in a sparse regime to the degree that weight coding with random connection fell behind connectivity coding with a fixed weight. Therefore, in a sparse regime, it is necessary to integrate representations by synaptic weights and connections, but how should we achieve such a representation? Theoretically speaking, we should choose a connection structure that minimizes the loss of information due to sparse connectivity. This can be achieved by minimizing the KL-divergence between the distribution of the external states estimated from the all-to-all network, and the distribution estimated from a given connection structure (i.e., $\underset{||C|{|}_{0}=\rho MN}{\mathrm{argmin}}{\langle D_{KL}\left[p\left(s^{t}|r_{X},C_{all}\right)||p\left(s^{t}|r_{X},C\right)\right]\rangle }_{r_{X}}$, see Section Optimality of Connectivity for details). However, this calculation requires combinatorial optimization, and local approximation is generally difficult (Donoho, 2006), thus expectedly the brain employs some heuristic alternatives. Experimental results indicate that synaptic connections and weights are often representing similar features. For example, the EPSP size of a connection in a clustered network is typically larger than the average EPSP size (Lefort et al., 2009; Perin et al., 2011), and a similar property is suggested to hold for interlayer connections (Yoshimura et al., 2005) (Ryan et al., 2015). Therefore, we could expect that by simply combining the weight coding and connectivity coding in the previous section, low performance at the sparse regime can be avoided, though convergence to the optimal solution is generally not achievable in this hybrid strategy even in the limit of infinitely many neurons. On the other hand, in the previous modeling studies, synaptic rewiring and resultant connection structure were often generated by cut-off algorithm in which a synapse is eliminated if the weight is smaller than the given criteria (Chechik et al., 1998; Navlakha et al., 2015). Thus, let us next compare the representation by combining the weight coding and connectivity coding (we call it as the dual coding below), with the cut-off coding strategy.

**Figure 3:**
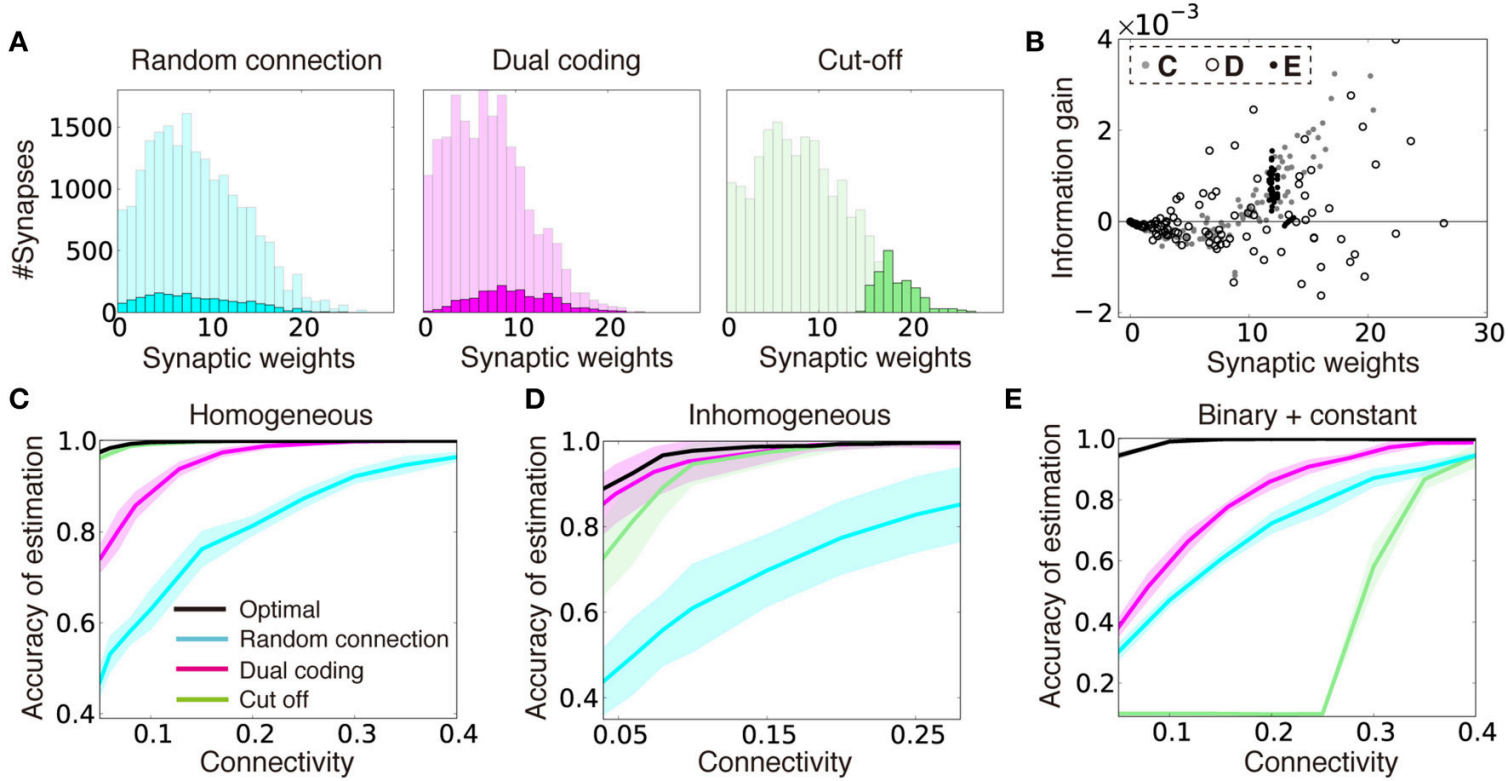
**Dual coding yields robust information representation compared to fixed random connections and cut-off strategy. (A)** Synaptic weight distributions in random connection (left), dual coding (middle), and cut-off (right) strategies in the model described **(C)**. Light colors represent possible connections (i.e., normalized distributions of optimal synaptic weights under all-to-all connections), while dark colors show the actual connections chosen under the different strategies. Connection probability was set at ρ = 0.1. **(B)** Relationships between the synaptic weight and the information gain per connection for three input configurations described in **(C–E)**. The open black circles were calculated with σ_*r*_ = 2.0 instead of σ_*r*_ = 4.0 for illustration purpose. **(C–E)** Comparisons of performance among different connection structure organizations for models with homogeneous input variability **(C)**, inhomogeneous input variability **(D)**, and the model with binary or constant input structures **(E)**. Note that black lines represent lower bounds for the optimal performance, but not the exact optimal solutions. In **(D)**, the means and standard deviations were calculated over 100 simulation trials instead of 10 due to intrinsic variability.

Figure 3A describes the synaptic weight distributions in the two strategies, as well as in random connection (see Section Dual Coding and Cut-Off Coding for details of the implementation) for the input structure used in Figure 3C. The light-colored distributions represent the normalized optimal synaptic weights for all-to-all connections, and the dark distributions represent the weights chosen from the light-colored distributions by each strategy. When connectivity coding and weight coding are combined (i.e., in the dual coding), connection probability becomes larger in proportion to its synaptic weight (Figure 3A middle), and the resultant distribution exhibits a broad distribution as observed in the experiments (Song et al., 2005; Ikegaya et al., 2013), whereas in the cut-off strategy, the weight distribution is concentrated at a non-zero value (Figure 3A, right). Intuitively, the cut-off strategy seems more selective and beneficial for inference. Indeed, in the original task, the cut-off strategy enabled near-optimal performance, though the dual coding also improved the performance compared to a randomly connected network (Figure 3C). However, under the presence of variability in the input layer, cut-off strategy is no longer advantageous. For instance, let us consider the case when noise amplitude σ_*X*_ is not constant but pre-neuron dependent. If the firing rate variability of input neuron *j* is given by σ_*X, j*_ ≡ σ_*X*_exp (2ζ_*j*_ log σ_*r*_) ∕ σ_*r*_, where ζ_*j*_ is a random variable uniformly sampled from [0, 1), and σ_*r*_ is the degree of variability, in an all-to-all network, optimal inference is still achieved by setting synaptic weights as $w_{ij}=q_{j\mu }\equiv \theta_{j\mu }∕\sigma_{X,j}^{2}$. On the contrary, in the sparse region, the performance is disrupted especially in the cut-off strategy, so that the dual coding outperformed the cut-off strategy (Figure 3D).

To further illustrate this phenomenon, let us next consider a case when a quarter of input neurons show a constant high response for all of the external states as ${\tilde{\theta }}_{j\mu }=\theta_{const},$ and the rest of input neurons show high response for randomly selected half of external states (i.e., $\mathrm{Pr}\left[{\tilde{\theta }}_{j\mu }=\theta_{high}\right]=\mathrm{Pr}\left[{\tilde{\theta }}_{j\mu }=\theta_{low}\right]=\frac{1}{2}$), where θ_*low*_ < θ_*high*_ < θ_*const*_, and $\theta_{j\mu }={\tilde{\theta }}_{j\mu }∕Z_{\mu }$ with the normalization factor $Z_{\mu }=r_{X}^{o}∕\sqrt{\overset{M}{\underset{j=1}{\sum }}{\tilde{\theta }}_{j\mu }∕M}$. Even in this case, $w_{ij}=q_{j\mu }\equiv \theta_{j\mu }∕\sigma_{X}^{2}$ is the optimal synaptic weights configuration in the all-to-all network, but if we create a sparse network with cut-off algorithm, the performance drops dramatically at certain connectivity, whereas in the dual coding, the accuracy is kept at some high levels even in the sparse connectivity (Figure 3E).

To get insights on why the dual coding is more robust against variability in the input layer, for three input configurations described above, we calculated the relationship between synaptic weight *w*_*ij*_ and the information gained by a single synaptic connection Δ*I*_*ij*_. Here, we defined the information gain Δ*I*_*ij*_ by the mean reduction in the KL divergence ${\langle D_{KL}\left[p\left(s^{t}|r_{X},C_{all}\right)||p\left(s^{t}|r_{X},C\right)\right]\rangle }_{r_{X}}$, achieved by adding one synaptic connection *c*_*ij*_ to a randomly connected network *C* (see Section Optimality of Connectivity for details). In the original model, Δ*I*_*ij*_ has nearly a linear relationship with the synaptic weight *w*_*ij*_ (gray points in Figure 3B), thus by simply removing the connections with small synaptic weights, a near-optimal connection structure was acquired (Figure 3C). On the other hand, when the input layer is not homogeneous, large synapses tend to have negative (black circles in Figure 3B) or zero (black points in Figure 3B) gains. As a result, the linear relationship between the weight and the information gain is no longer observed. Thus, in these cases, the dual coding is less likely to be disrupted by non-beneficial connections.

Although our consideration here is limited to a specific realization of synaptic weights, in general, it is difficult to represent the information gain by locally acquired synaptic weight, so we could expect that the cut-off strategy is not the optimal connectivity organization in many cases.

### Local hebbian learning of the dual coding

The argument in the previous section suggest that, by combining the weight coding and connectivity coding, the network can robustly perform inference especially in sparsely connected regions. However, in the previous sections, a specific connection and weight structure were given *a priori*, although structures in local neural circuits are expected to be obtained with local weight plasticity and wiring plasticity. Thus, we next investigate whether dual coding can be achieved through a local unsupervised synaptic plasticity rule.

Let us first consider learning of synaptic weights. In order to achieve the weight coding, synaptic weight *w*_*ij*_ should converge to $w_{ij}=q_{j\mu }∕\sigma_{X}^{2}\overset{-}{\rho }=\langle r_{X,j}^{t}r_{Y,i}^{t}∕\left(\sigma_{X}^{2}\overset{-}{\rho }r_{Y,i}^{t}\right)\rangle$ when output neuron *i* represents external state μ, and $\overset{-}{\rho }$ represents the mean connectivity of the network. Thus, synaptic weight change $\Delta w_{ij}=w_{ij}^{t+1}-w_{ij}^{t}$ is given as:

(2) $$\Delta w_{ij}=\left(\eta_{X}/\gamma \right)\left(r_{Y,i}^{t}\left[r_{X,j}^{t}-\sigma_{X}^{2}\bar{\rho }w_{ij}\right]\;+\;b_{h}\left[r_{Y}^{o}/N-r_{Y,i}^{t}\right]\right).$$

**Figure 4:**
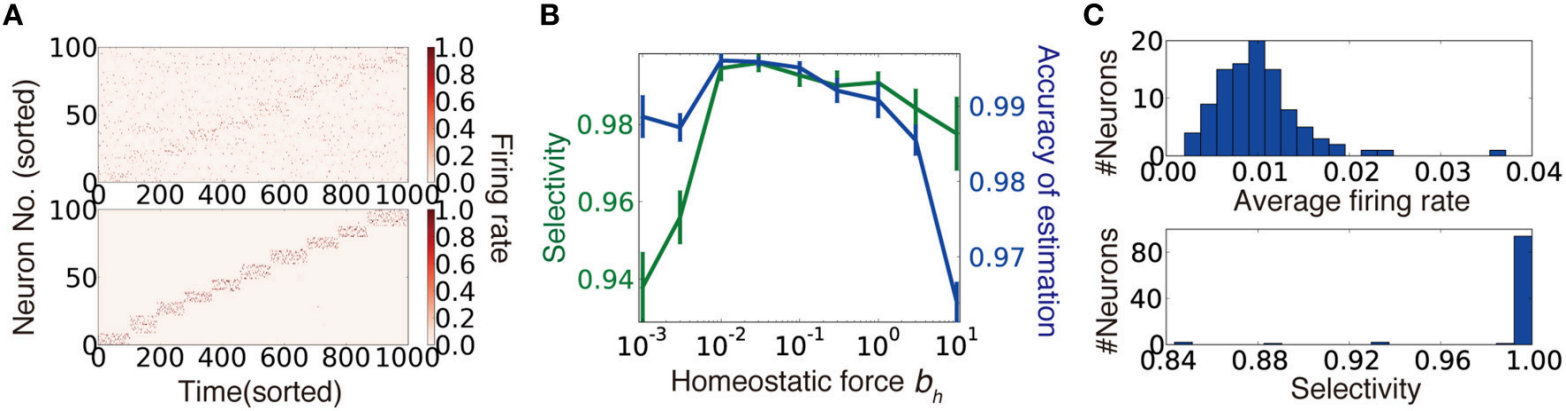
**Synaptic weight learning on random connection structures**. **(A)** An example of output neuron activity before (top) and after (bottom) synaptic weight learning calculated at connectivity ρ = 0.4. A diagonal line faintly observed in the upper panel reflects bias due to finite size effect (see Section Accuracy of Estimation for details) **(B)** Selectivity of output neurons and accuracy of estimation at various strengths of homeostatic plasticity at ρ = 0.4. Selectivity was defined as $\sum_{s^{t}=\mu }r_{Y,i}^{t}/\sum tr_{Y,i}^{t}$ for *i* ∈ Ω_μ_. **(C)** Histogram of average firing rates of output neurons (top), and selectivity of each neuron calculated for the simulation depicted in **(A)**.

The second term is the homeostatic term heuristically added to constrain the average firing rates of output neurons (Turrigiano and Nelson, 2004). Note that the first term corresponds to stochastic gradient descending on $D_{KL}\left[p^{*}\left(r_{X}^{t}\right)||p\left(r_{X}^{t}|C,W\right)\right]$, because the weight coding approximates the optimal representation by synaptic weights (Nessler et al., 2013; see Section Synaptic Weight Learning for details). We performed this unsupervised synaptic weight learning on a randomly connected network. When the connectivity is sufficiently dense, the network successfully acquired a suitable representation (Figure 4A). Especially under a sufficient level of homeostatic plasticity (Figure 4B), the average firing rate showed a narrow unimodal distribution (Figure 4C, top), and most of the output neurons acquired selectivity for one of external states (Figure 4C, bottom).

We next investigated the learning of connection structures by wiring plasticity. Unlike synaptic weight plasticity, it is not yet well understood how we can achieve functional connection structure with local wiring plasticity. In particular, rapid rewiring may disrupt the network structure, and possibly worsen the performance (Chechik et al., 1998). Thus, let us first consider a simple rewiring rule, and discuss the biological correspondence later. Here, we introduced a variable ρ_*ij*_, for each combination *(i,j)* of presynaptic neuron *j* and postsynaptic neuron *i*, which represents the connection probability. If we randomly create a synaptic connection between neuron *(i,j)* with probability ρ_*ij*_/τ_*c*_ and eliminate it with probability (1−ρ_*ij*_)/τ_*c*_, on average there is a connection between neuron *(i,j)* with probability ρ_*ij*_, when the maximum number of synaptic connections is bounded by 1. In this way, the total number of synaptic connections is kept constant on average, without any global regulation mechanism. Throughout the paper, when a new spine is created, we set its initial synaptic weight as $w_{ij}=\left(1+\sigma_{w}^{init}\zeta \right)∕\gamma$, not by the value calculated from Equation (2), for biological plausibility.

From a similar argument done for synaptic weights, the learning rule for connection probability ρ_*ij*_ is derived as:

(3) $$\Delta \rho_{ij}=\eta_{\rho }r_{Y,i}^{t}\left[r_{X,j}^{t}-\sigma_{X}^{2}\rho_{ij}w_{o}\right],$$

where *w*_*o*_ is the expected mean synaptic weight (Section Synaptic Connection Learning). Under this rule, the connection probabilities converge to the connectivity coding. Moreover, although this rule does not maximize the transfer entropy of the connections, direction of learning is on average close to the direction of the stochastic gradient on transfer entropy. Therefore, the above rule does not reduce the transfer entropy of the connection on average (see Section Dual Hebbian Rule and Estimated Transfer Entropy).

**Figure 5:**
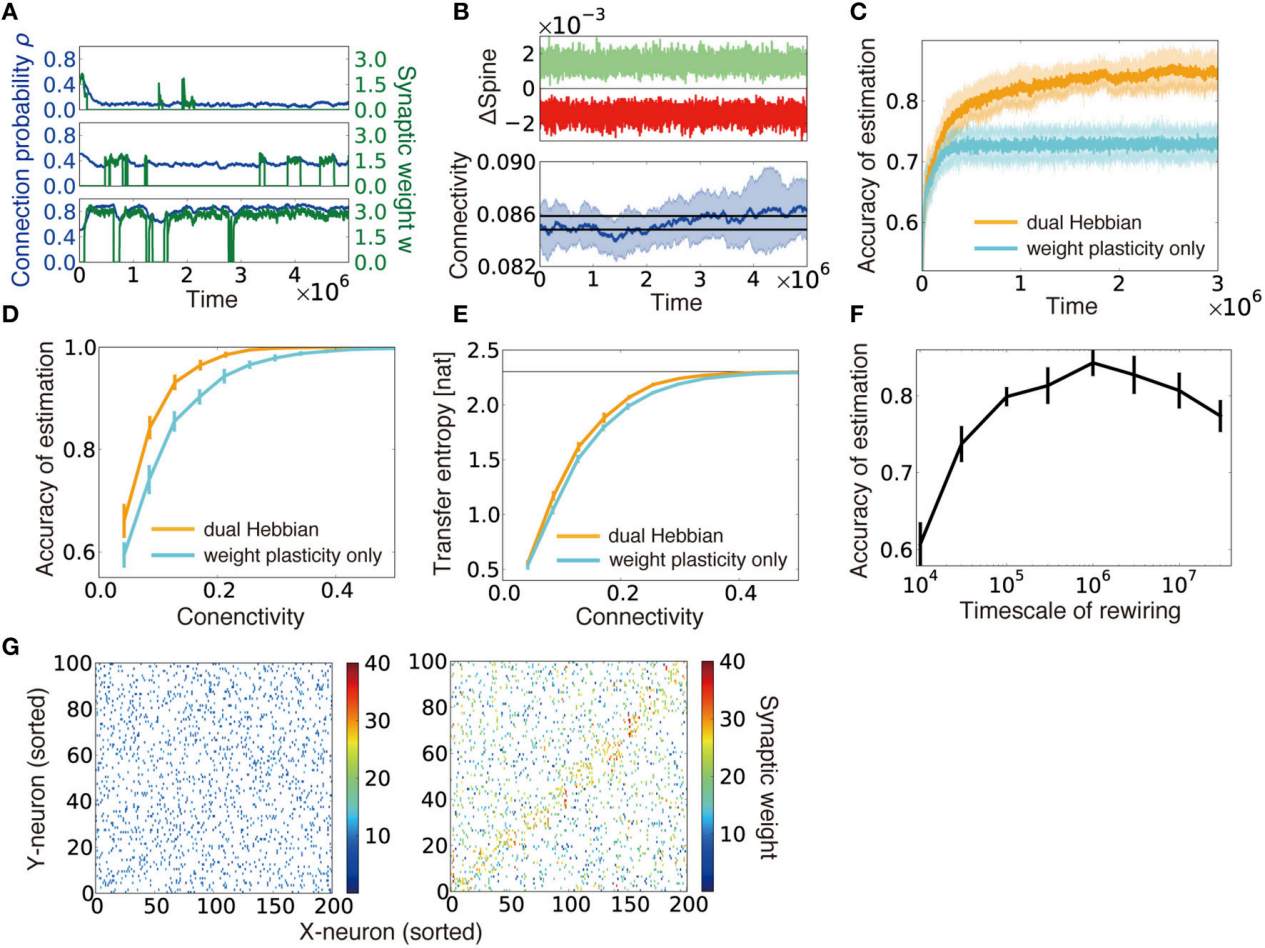
**Dual Hebbian learning for synaptic weights and connections**. **(A)** Examples of spine creation and elimination. In all three panels, green lines show synaptic weights, and blue lines are connection probability. When there is not a synaptic connection between two neurons, the synaptic weight becomes zero, but the connection probability can take a non-zero value. Simulation was calculated at ρ = 0.48, η_ρ_ = 0.001, and τ_*c*_ = 10^5^. **(B)** Change in connectivity due to synaptic elimination and creation. Number of spines eliminated (red) and created (green) per unit time was balanced (top). As a result, connectivity did not appreciably change due to rewiring (bottom). Black lines in the bottom graph are the mean connectivity at γ = 0.1 and γ = 0.101 in the model without rewiring. **(C)** Accuracy of estimation for the model with/without wiring plasticity. For the dual Hebbian model, the sparseness parameter was set as γ = 0.1, whereas γ = 0.101 was used for the weight plasticity model to perform comparisons at the same connectivity (see **B**). **(D,E)** Comparison of the performance **(D)** and the maximum estimated transfer entropy **(E)** after learning between the dual Hebbian model and the model implemented with synaptic plasticity only at various degrees of connectivity. Horizontal line in **(E)** represents the total information log_e_*p*. **(F)** Accuracy of estimation with various timescales for rewiring τ_*c*_. Note that the simulation was performed only for 5 × 10^6^ time steps, and the performance did not converge for the model with a longer timescale. **(G)** Synaptic weight matrices before (left) and after (right) learning. Both X-neurons (input neuron) and Y-neurons (output neurons) were sorted based on their preferred external states.

Figure 5A shows the typical behavior of ρ_*ij*_ and *w*_*ij*_ under combination of this wiring rule (Equation 3) and the weight plasticity rule described in Equation (2) (we call this combination as the dual Hebbian rule because both Equations 2 and 3 have Hebbian forms). When the connection probability is low, connections between two neurons are rare, and, even when a spine is created due to probabilistic creation, the spine is rapidly eliminated (Figure 5A, top). In the moderate connection probability, spine creation is more frequent, and the created spine survives longer (Figure 5A, middle). When the connection probability is high enough, there is almost always a connection between two neurons, and the synaptic weight of the connection is large because synaptic weight dynamics also follow a similar Hebbian rule (Figure 5A, bottom).

We implemented the dual Hebbian rule in our model and compared the performance of the model with that of synaptic weight plasticity on a fixed random synaptic connection structure. Because spine creation and elimination are naturally balanced in the proposed rule (Figure 5B, top), the total number of synaptic connections was nearly unchanged throughout the learning process (Figure 5B, bottom). As expected, the dual Hebbian rule yielded better performance (Figures 5C,D) and higher estimated transfer entropy than the corresponding weight plasticity only model (Figure 5E). This improvement was particularly significant when the frequency of rewiring was in an intermediate range (Figure 5F). When rewiring was too slow, the model showed essentially the same behavior as that in the weight plasticity only model, whereas excessively frequent probabilistic rewiring disturbed the connection structure. Although a direct comparison with experimental results is difficult, the optimal rewiring timescale occurred within hours to days, under the assumption that firing rate dynamics (Equation 1) are updated every 10–100 ms. Initially, both connectivity and weights were random (Figure 5G, left), but after the learning process, the diagonal components of the weight matrix developed relatively larger synaptic weights, and, at the same time, denser connectivity than the off-diagonal components (Figure 5G, right). Thus, through dual Hebbian learning, the network can indeed acquire a connection structure that enables efficient information transmission between two layers; as a result, the performance improves when the connectivity is moderately sparse (Figures 5D,E). Although the performance was slightly worse than that of a fully-connected network, synaptic transmission consumes a large amount of energy (Sengupta et al., 2013), and synaptic connection is a major source of noise (Faisal et al., 2008). Therefore, it is beneficial for the brain to achieve a similar level of performance using a network with fewer connections.

### Connection structure can acquire constant components of stimuli and enable rapid learning

**Figure 6:**
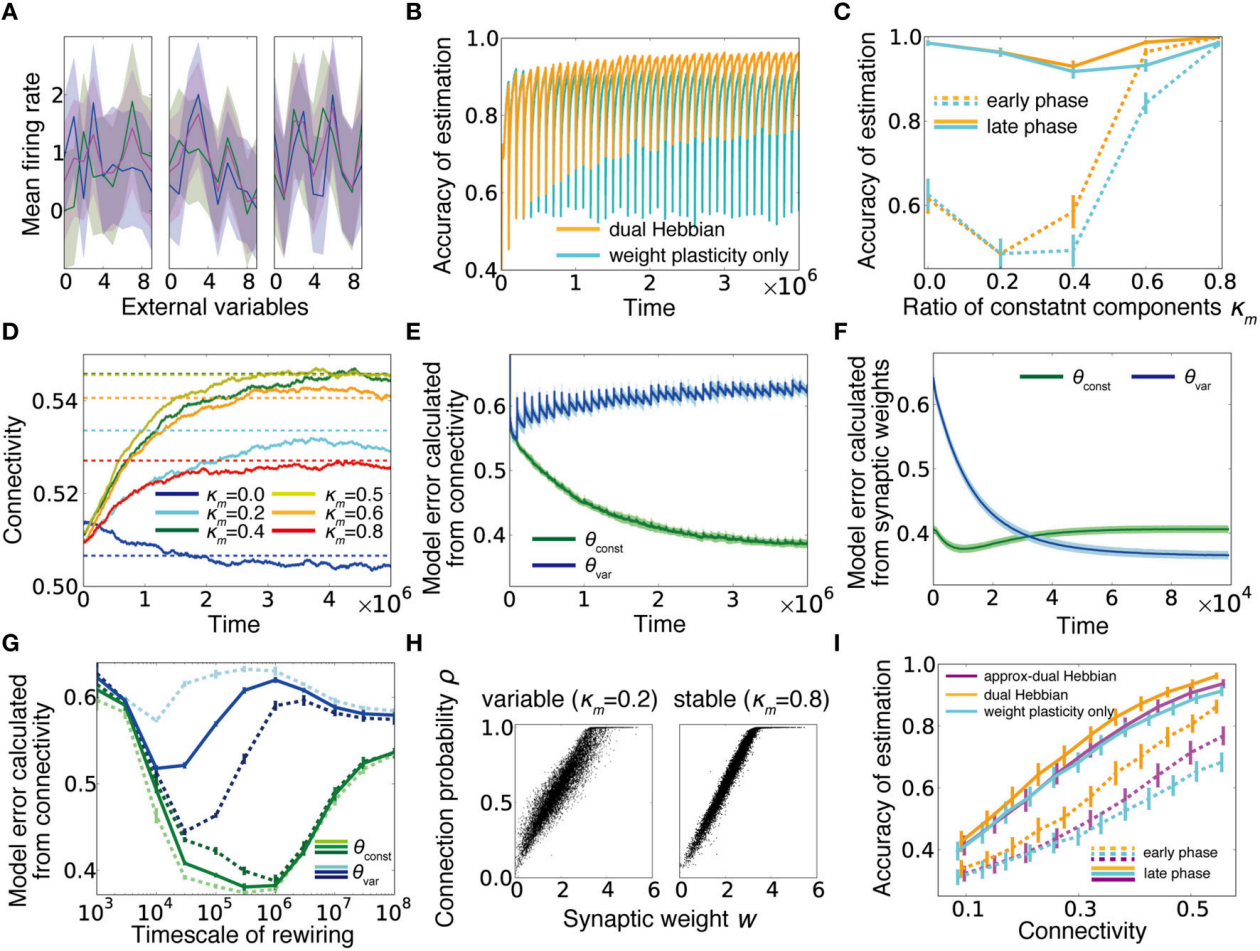
**Dual Hebbian learning under a dynamic environment**. **(A)** Examples of input neuron responses. Blue lines represent the constant components θ_*const*_, green lines show the variable components θ_*var*_, which are updated at every *T*_2_ time steps by sampling a new input structure from a truncated Gaussian distribution (see Section Gaussian Model), and magenta lines are the total external models θ calculated from the normalized sum. **(B)** Learning curves for the model with or without wiring plasticity, when the variable components change every 10^5^ time steps. **(C)** Accuracy of estimation for various ratios of constant components. Early phase performance was calculated from the activity within 10,000 steps after the variable component shift, and the late phase performance was calculated from the activity within 10,000 steps before the shift. As in **(B)**, orange lines represent the dual Hebbian model, and cyan lines are for the model with weight plasticity only. **(D)** Trajectories of connectivity change. Connectivity tends to increase slightly during learning. Dotted lines are mean connectivity at (κ_*m*_, γ) = (0.0, 0.595), (0.2, 0.625), (0.4, 0.64), (0.5, 0.64), (0.6, 0.635), and (0.8, 0.620). In **(C)**, these parameters were used for the synaptic plasticity only model, whereas γ was fixed at γ = 0.6 for the dual Hebbian model. **(E,F)** Model error calculated from connectivity **(E)** and synaptic weights **(F)**. Note that the timescale of **(F)** is the duration in which the variable component is constant, not the entire simulation (i.e., the scale of x-axis is 10^4^ not 10^6^). **(G)** Model error calculated from connectivity for various rewiring timescales τ_*c*_. For a large τ_*c*_, the learning process does not converge during the simulation. Dotted lines are results for *T*_2_ = 3 × 10^4^ (pale lines), and *T*_2_ = 3 × 10^5^ (dark lines). Note that the splitting point of θ_*const*_ and θ_*var*_ shifts for the left/right sides in the pale/dark lines. **(H)** Relationship between synaptic weight *w* and connection probability ρ at the end of learning. When the external model is stable, *w* and ρ have a more linear relationship than that for the variable case. **(I)** Comparison of performances among the model without wiring plasticity (cyan), the dual Hebbian model (orange), the approximated model (magenta).

We have shown that the dual coding by synaptic weights and connections robustly helps computation in a sparsely connected network, and the desirable weight and connectivity structures are naturally acquired through the dual Hebbian rule. Although we were primary focused on sparse regions, the rule potentially provides some beneficial effects even in densely connected networks. To consider this issue, we extended the previous static external model to a dynamic one, in which at every interval *T*_2_, response probabilities of input neurons partly change. If we define the constant component as θ_*const*_ and the variable component as θ_*var*_, then the total model becomes $\theta_{j\mu }=\frac{1}{Z}\left[\kappa_{m}\theta_{j\mu }^{const}+\left(1-\kappa_{m}\right)\theta_{j\mu }^{\text{var}}\right]$, where the normalization term is given as $\frac{1}{MZ^{2}}\overset{M}{\underset{j=1}{\sum }}{\left[\kappa_{m}\theta_{j\mu }^{const}+\left(1-\kappa_{m}\right)\theta_{j\mu }^{\text{var}}\right]}^{2}={\left(r_{X}^{o}\right)}^{2}$ (Figure 6A). Below, we updated θ_*var*_ at every *T*_2_ = 10^5^ time steps. In this setting, when the learning was performed only with synaptic weights based on fixed random connections, although the performance rapidly improved, every time a part of the model changed, the performance dropped dramatically and only gradually returned to a higher level (cyan line in Figure 6B). By contrast, under the dual Hebbian learning rule, the performance immediately after the model shift (i.e., the performance at the trough of the oscillation) gradually increased, and convergence became faster (Figures 6B,C), although the total connectivity stayed nearly the same (Figure 6D). After learning, the synaptic connection structure showed a higher correlation with the constant component than with the variable component (Figure 6E; see Section Model Error). By contrast, at every session, synaptic weight structure learned the variable component better than it learned the constant component (Figure 6F). The timescale for synaptic rewiring needed to be long enough to be comparable with the timescale of the external variability *T*_2_ to capture the constant component. Otherwise, connectivity was also strongly modulated by the variable component of the external model. In Figure 6G, lines represent the model errors for three different values of *T*_2_ at various timescales of rewiring. In addition, we find that the rewiring timescale should be in an intermediate range as also observed in Figure 5F. After sufficient learning, the synaptic weight *w* and the corresponding connection probability ρ roughly followed a linear relationship (Figure 6H). Remarkably, some synapses developed connection probability ρ = 1, meaning that these synapses were almost permanently stable because the elimination probability (1−ρ)/τ_*c*_ became nearly zero.

### Approximated dual hebbian learning rule reconciles with experimentally observed spine dynamics

Our results up to this point have revealed functional advantages of dual Hebbian learning. In this last section, we investigated the correspondence between the experimentally observed spine dynamics and the proposed rule. To this end, we first studied whether a realistic spine dynamics rule approximates the proposed rule, and then examined if the rule explains the experimentally known relationship between synaptic rewiring and motor learning (Xu et al., 2009; Yang et al., 2009).

Previous experimental results suggest that a small spine is more likely to be eliminated (Yasumatsu et al., 2008; Kasai et al., 2010), and spine size often increases or decreases in response to LTP or LTD respectively, with a certain delay (Matsuzaki et al., 2004; Wiegert and Oertner, 2013). In addition, though spine creation is to some extent influenced by postsynaptic activity (Knott et al., 2006; Yang et al., 2014), the creation is expected to be more or less a random process (Holtmaat and Svoboda, 2009). Thus, changes in the connection probability can be described as

(4) $$\rho_{ij}^{t}=\{\begin{array}{l} \rho_{ij}^{t\;-\;1}+\eta_{\rho }\left[\gamma^{2}w_{ij}-\rho_{ij}^{t\;-\;1}\right]\;\left(\text{if }c_{ij}=1\right)\\ \gamma^{2}w_{o}\;\left(\text{if }c_{ij}=0\right). \end{array}$$

**Figure 7:**
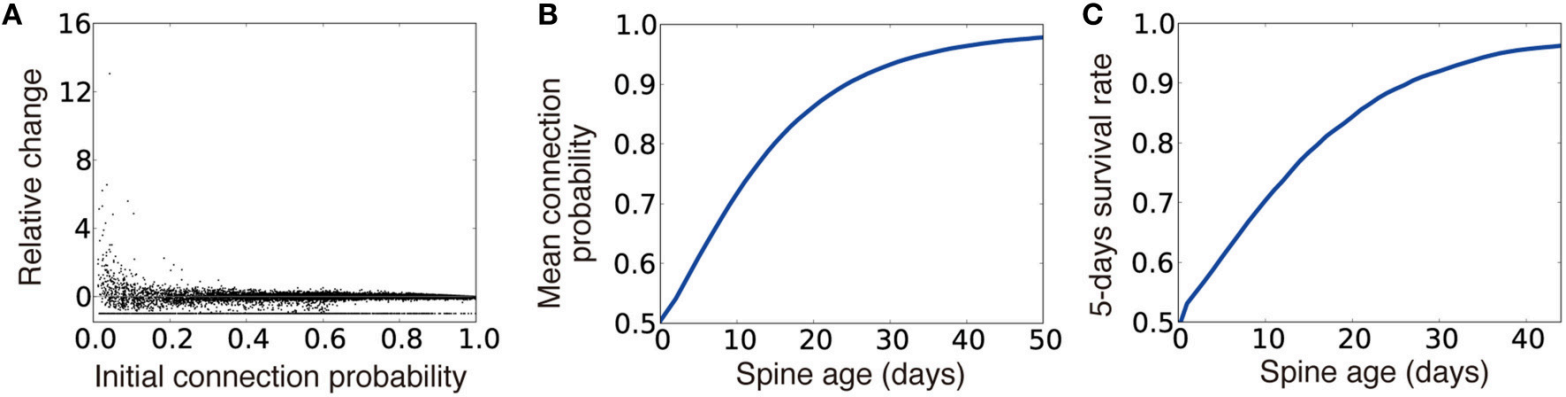
**Spine dynamics of the approximated dual Hebbian model. (A)** Relative change of connection probability in 10^5^ time steps. If the initial connection probability is low, the relative change after 10^5^ time steps has a tendency to be positive, whereas spines with a high connection probability are more likely to show negative changes. The line at the bottom represents eliminated spines (i.e., relative change = −1). **(B,C)** Relationships between spine age and the mean connection probability **(B)** and the 5-days survival rate **(C)**. Consistent with the experimental results, survival rate is positively correlated with spine age. Five days survival rate was calculated by regarding 10^5^ time steps as 1 day.

By combining this rule and the Hebbian weight plasticity described in Equation (2), the dynamics of connection probability well replicated the experimentally observed spine dynamics (Yasumatsu et al., 2008; Kasai et al., 2010; Figures 7A–C). Moreover, the rule outperformed the synaptic weight only model in the inference task, although the rule performed poorly compared to the dual Hebbian rule due to the lack of activity dependence in spine creation (magenta line in Figure 6I). This result suggests that plasticity rule by Equations (2) and (4) well approximates the dual Hebbian rule (Equations 2+3). This is because, even if the changes in the connection probability are given as a function of synaptic weight as in Equation (4), as long as the weight plasticity rule follows Equation (2), wiring plasticity indirectly shows a Hebbian dependency for pre- and postsynaptic activities as in the original dual Hebbian rule (Equation 3). As a result, the approximated rule gives a good approximation of the original dual Hebbian rule.

**Figure 8:**
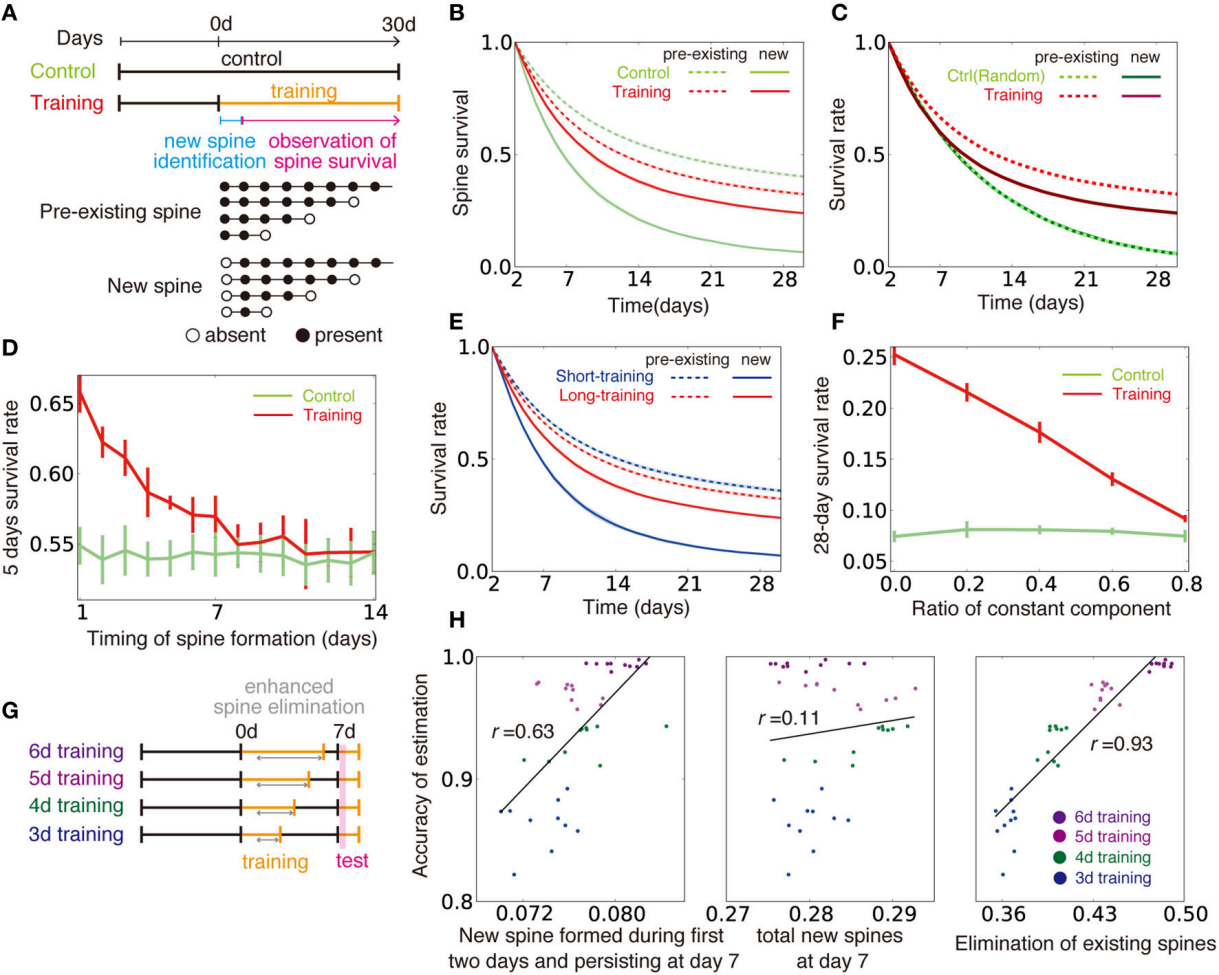
**Influence of training on spine dynamics. (A)** Schematic diagrams of the simulation protocols for **(B–F)**, and examples of spine dynamics for pre-existing spines and new spines. **(B)** Spine survival rates for control and training simulations. Dotted lines represent survival rates of pre-existing spines (spines created before day 0 and existing on day 2), and solid lines are new spines created between day 0 and day 2. **(C)** Spine survival rates when control was modeled with random Gaussian inputs $r_{X,j}^{t}=0.75\mu_{M}+\sqrt{\sigma_{x}^{2}+\sigma_{M}^{2}}\zeta_{j}^{t}$, instead of a pattern {θ_*ctrl*_}. Note that light-green line is hidden under dotted green line, because two lines show nearly identical dynamics. **(D)** The 5-day survival rate of spines created at different stages of learning. **(E)** Spine survival rates for short-training (2 d) and long-training (30 d) simulations. Pre-existing and new spines were defined as in **(A,B)**. **(F)** Effect of similarity between the control condition and training on the new spine survival rate. The value of κ_*m*_ was changed as in Figure 6C to alter the similarity between the two conditions. Note that κ_*m*_ = 0 in **(A–E,G,H)**. **(G, H)** Relationships between creation and elimination of spines and task performance. Performance was calculated from the activity within 2000–7000 time steps after the beginning of the test phase (see Section Spine Dynamics for details). In the simulation, the synaptic elimination was increased fivefold from day 1 to the end of training.

We next applied this approximated learning rule to motor learning tasks. The primary motor cortex has to adequately read-out motor commands based on inputs from pre-motor regions (Salinas and Romo, 1998; Sul et al., 2011). In addition, the connection from layer 2/3 to layer 5 is considered to be a major pathway in motor learning (Masamizu et al., 2014). Thus, we hypothesized that the input and output layers of our model can represent layers 2/3 and 5 in the motor cortex. We first studied the influence of training on spine survival (Xu et al., 2009; Figure 8A). To compare with experimental results, below we regarded 10^5^ time steps as 1 day, and described the training and control phases as two independent external models θ_*ctrl*_ and θ_*train*_. We assumed that the corresponding neural circuits are already tuned and actively employed for processing certain structured inputs, even in the control animal, so that training is actually a retraining on a new input structure. Under this assumption, survival ratio of newly created and pre-existing spines exhibits a large difference, as observed in the experiment (Figure 8B). However, the difference is difficult to replicate when the control is modeled as a blank slate with unstructured inputs (Figure 8C). As observed for the control case, newly created spines were less stable than pre-existing spines, also in the training case (solid lines vs. dotted lines in Figure 8B), because older spines tended to have a larger connection probability (Figure 7B). Nevertheless, continuous training turned pre-existed spines less stable and new spines more stable than their respective counterparts in the control case (red lines vs. lime lines in Figure 8B). The 5-day survival rate of a spine was higher for spines created within a couple of days from the beginning of training compared with spines in the control case, whereas the survival rate converged to the control level after several days of training (Figure 8D). Our model also replicates the effect of varying training duration on spine stability (Yang et al., 2009). When training on new input structure θ_*training*_ was rapidly terminated and the inputs structure went back to the control θ_*ctrl*_, newly formed spines became less stable than those undergoing training on new input structure for a long period continuously (Figure 8E). In addition, we found that θ_*ctrl*_ and θ_*train*_ need to be independent to observe the above results (Figure 8F).

We next considered the relationship between spine dynamics and task performance (Yang et al., 2009). For this purpose, we compared task performance at the beginning of the test period among simulations with various training lengths (Figure 8G). Here, we assumed that spine elimination was enhanced during continuous training, as is observed in experiments (Xu et al., 2009; Yang et al., 2009). The performance was positively correlated with both the survival rate at day 7 of new spines formed during the first 2 days, and the elimination rate of existing spines (left and right panels of Figure 8H; see Section Spine Dynamics for details). By contrast, the performance was independent from the total ratio of newly formed spines from day 0 to 6 (middle panel of Figure 8H). These results demonstrate that complex spine dynamics are well described by the approximated dual Hebbian rule, suggesting that the brain uses a dual learning mechanism.

## Discussion

In this study, we first analyzed how random connection structures impair performance in sparsely connected networks by analyzing the change in signal variability and the transfer entropy in the weight coding and the connectivity coding strategies (Figure 2). Subsequently, we showed that connection structures created by the cut-off strategy are not beneficial under the presence of input variability, due to lack of positive correlation between the information gain and weight of synaptic connections (Figure 3). Based on these insights, we proposed that the dual coding by weight and connectivity structures as a robust representation strategy, then demonstrated that the dual coding is naturally achieved through dual Hebbian learning by synaptic weight plasticity and wiring plasticity (Figures 4, 5). We also revealed that, even in a densely connected network in which synaptic weight plasticity is sufficient in terms of performance, by encoding the time-invariant components with synaptic connection structure, the network can achieve rapid learning and robust performance (Figure 6). Even if spine creation is random, the proposed framework still works effectively, and the approximated model with random spine creation is indeed sufficient to reproduce various experimental results (Figures 7, 8).

### Model evaluation

Spine dynamics depend on the age of the animal (Holtmaat et al., 2005), the brain region (Zuo et al., 2005), and many molecules play crucial roles (Kasai et al., 2010; Caroni et al., 2012), making it difficult for any theoretical models to fully capture the complexity. Nevertheless, our simple mathematical model replicated many key features (Yasumatsu et al., 2008; Xu et al., 2009; Yang et al., 2009; Kasai et al., 2010). For instance, small spines often show enlargement, while large spines are more likely to show shrinkage (Figure 7A). Older spines tend to have a large connection probability, which is proportional to spine size (Figure 7B), and they are more stable (Figure 7C). In addition, training enhances the stability of newly created spines, whereas it degrades the stability of older spines (Figure 8B).

### Experimental prediction

In the developmental stage, both axon guidance (Munz et al., 2014) and dendritic extension (Matsui et al., 2013) show Hebbian-type activity dependence, but in the adult cortex, both axons and dendrites seldom change their structures (Holtmaat and Svoboda, 2009). Thus, although recent experimental results suggest some activity dependence for spine creation (Knott et al., 2006; Yang et al., 2014), it is still unclear to what extent spine creation depends on the activity of presynaptic and postsynaptic neurons. Our model indicates that in terms of performance, spine creation should fully depend on both presynaptic and postsynaptic activity (Figure 6I). However, we also showed that it is possible to replicate a wide range of experimental results on spine dynamics without activity-dependent spine creation (Figure 8).

Furthermore, whether or not spine survival rate increases through training is controversial (Xu et al., 2009; Yang et al., 2009). Our model predicts that the stability of new spines highly depends on the similarity between the new task and control behavior (Figure 8F). When the similarity is low, new spines created in the new task are expected to be more stable than those created in the control case, because the synaptic connection structure would need to be reorganized. By contrast, when the similarity is high, the stability of the new spines would be comparable to that of the control.

### Related studies

Previous theoretical studies revealed candidate rules for spine creation and elimination (Deger et al., 2012; Zheng et al., 2013; Fauth et al., 2015), yet their functional benefits were not fully clarified in those studies. Some modeling studies considered the functional implications of synaptic rewiring (Poirazi and Mel, 2001) or optimality in regard to benefit and wiring cost (Chen et al., 2006), but the functional significance of synaptic plasticity and the variability of EPSP size were not considered in those models. In comparison, our study revealed functional roles of wiring plasticity that cooperates with synaptic weight plasticity and obeys local unsupervised rewiring rules. In addition, we extended the previous results on single-spine information storage and synaptic noise (Varshney et al., 2006) into a network, and provided a comparison with experimental results (Figure 2E).

Previous studies on associative memory models found the cut-off coding as the optimal strategy for maximizing the information capacity per synapse (Chechik et al., 1998; Knoblauch et al., 2010). Our results suggest that the above result is the outcome of the tight positive correlation between the information gain and synaptic weight in associative memory systems, and not generally applicable to other paradigms (Figures 3B,C). In addition, although cut-off strategy did not yield biologically plausible synaptic weight distributions in our task setting (Figure 3A, right), in perceptron-based models, this unrealistic situation can be avoided by tuning the threshold of neural dynamics (Brunel et al., 2004; Sacramento et al., 2015). Especially, cut-off strategy may provide a good approximation for developmental wiring plasticity (Ko et al., 2013), though the algorithm is not fully consistent with wiring plasticity in the adult animals.

Finally, our model provides a biologically plausible interpretation for multi-timescale learning processes. It was previously shown that learning with two synaptic variables on different timescales is beneficial under a dynamically changing environment (Fusi et al., 2007). In our model, both fast and slow variables played important roles, whereas in previous studies, only one variable was usually more effective than others, depending on the task context.

## Methods

### Model

#### Model dynamics

We first define the model and the learning rule for general exponential family, and derive equations for two examples (Gaussian and Poisson). In the task, at every time *t*, one hidden state *s*^*t*^ is sampled from prior distribution *p*(*s*^*t*^) (see Table 1 for the definitions of variables and parameters). Neurons in the input layer show stochastic response $r_{X,j}^{t}$ that follows probabilistic distribution *f* ($r_{X,j}^{t}$ |*s*^*t*^ = μ):

(5) $$f(r_{X,j}^{t}|s^{t}=\mu )\equiv \exp\left[h(\theta_{j\mu })g(r_{X,j}^{t})-A(\theta_{j\mu })+B(r_{X,j}^{t})\right].$$

From these input neuron activities, neurons in output layer estimate the hidden variables. Here we assume maximum likelihood estimation for decision making unit, as the external state is a discrete variable. In this framework, in order to detect the hidden signal, firing rate of neuron *i* should be proportional to posterior

(6) $$r_{Y,i}^{t}\propto \mathrm{Pr}\left[s^{t}=\sigma_{i}|r_{X}^{t}\right].$$

where σ_*i*_ represents the index of the hidden variable preferred by output neuron *i* (Beck et al., 2008; Lochmann and Deneve, 2011). For instance, if output neuron *i* is selective for the hidden variable *s*^*t*^ = μ, then σ_*i*_ = μ. Note that {*r*_*X, j*_} represent firing rates of input neurons, whereas {*r*_*Y, i*_} represent the rates of output neurons. Due to Bayes rule, estimation of *s*^*t*^ is given by,

(7) $$\begin{array}{l} \log p(s^{t}=\mu |r_{X}^{t})=\sum_{j\;=\;1}^{M}\log p(r_{X,j}^{t}|s^{t}=\mu )+\log p(s^{t}=\mu )\\ \;-\log p(r_{X}^{t})\\ \;=\sum_{j\;=\;1}^{M}\left[q_{\mu j}g(r_{X,j}^{t})-\alpha (q_{\mu j})+B(r_{X,j}^{t})\right]\\ \;+\log p(s^{t}=\mu )-\log p(r_{X}^{t}), \end{array}$$

where *q*_*jμ*_ ≡ *h*(θ_*jμ*_), $\alpha \left(q_{j\mu }\right)\equiv A\left(h^{-1}\left(q_{j\mu }\right)\right)$. If we assume the uniformity of hidden states as log *p*(*s*^*t*^ = μ) : *const*, and $\frac{1}{M}\overset{M}{\underset{j=1}{\sum }}\alpha \left(q_{j\mu }\right)=\alpha_{o}$, the equation above becomes

$$\log p(s^{t}=\mu |r_{X}^{t})=\sum_{j\;=\;1}^{M}\left[q_{\mu j}g(r_{X,j}^{t})+B(r_{X,j}^{t})\right]-\log p(r_{X}^{t})+\text{const}.$$

To achieve neural implementation of this inference problem, let us consider a neural dynamics in which the firing rates of output neurons follow,

(8) $$r_{Y,i}^{t}=r_{Y}^{o}\exp\left[\sum_{j\;=\;1}^{M}c_{ij}\left(w_{ij}g(r_{X,j}^{t})-h_{w}\right)-I_{inh}^{t}\right],$$

where,

$$I_{inh}^{t}\equiv \log\left[\sum_{i=1}^{N}\exp\left(\sum_{j\;=\;1}^{M}c_{ij}\left[w_{ij}g(r_{X,j}^{t})-h_{w}\right]\right)\right],$$

and *h*_*w*_ is the threshold. If connection is all-to-all, *w*_*ij*_ = *q*_*jμ*_ gives optimal inference, because

(9) $$\frac{r_{Y,i}^{t}}{r_{Y}^{o}}=\frac{\exp\left[\sum_{j}q_{j\mu }g(r_{X,j}^{t})\right]}{\sum_{v}\exp\left[\sum_{j}q_{jv}g(r_{X,j}^{t})\right]}=p(s^{t}=\mu |r_{X}^{t})$$

Note that *h*_*w*_ is not necessary to achieve optimal inference, however, under a sparse connection, *h*_*w*_ is important for reducing the effect of connection variability. In this formalization, even in non-all-to-all network, if the sparseness of connectivity stays in reasonable range, near-optimal inference can be performed for arbitrary feedforward connectivity by adjusting synaptic weight to *w*_*ij*_ = *w*_μ*j*_ ≡ *q*_*jμ*_ ∕ ρ_μ*j*_ where $\rho_{\mu j}=\frac{1}{\left|\Omega_{\mu }\right|}\underset{i\in \Omega_{\mu }}{\sum }c_{ij}$, and Ω_μ_ is the set of output neurons selective for external state μ.

#### Weight coding and connectivity coding

Let us first consider the case when the connection probability is constant (i.e., ρ_*ij*_ = ρ). By substituting ρ_*ij*_ = ρ into the above equations, *c* and *w* are given with Pr [*c*_*ij*_ = 1] = ρ and *w*_*ij*_ = *w*_μ*j*_ = *q*_*jμ*_ ∕ ρ, where the mean connectivity is given as $\rho =\gamma \overset{-}{q}$, and $\overset{-}{q}$ is the average of the normalized mean response *q*_*jμ*_ (i.e., $\overset{-}{q}=\frac{1}{Mp}\underset{j}{\sum }\underset{\mu }{\sum }q_{j\mu }$). Parameter γ is introduced to control the sparseness of connections, and here we assumed that neuron *i* represents the external state $\mu =floor\left(\frac{p\times i}{N}\right)$ (i.e., if $\frac{\mu N}{p}<i\leq \frac{\left(\mu +1\right)N}{p}$, output neuron *i* represents the state μ). Under this configuration, the representation is solely achieved by the synaptic weights, thus we call this coding strategy as the weight coding.

On the other hand, if the synaptic weight is kept at a constant value, the representation is realized by synaptic connection structure (i.e., connectivity coding). In this case, the model is given by Pr [*c*_*ij*_ = 1] = ρ_μ*j*_ and *w*_*ij*_ = *w*_μ*j*_ = 1∕γ, where ρ_μ*j*_ = min(γ*q*_*jμ*_, 1).

#### Dual coding and cut-off coding

By combining the weight coding and connectivity coding described above, the dual coding is given as *w*_*ij*_ = *w*_μ*j*_ = *q*_*jμ*_∕ρ, Pr [*c*_*ij*_ = 1] = ρ_μ*j*_, ρ_μ*j*_ = min(γ*q*_*jμ*_, 1), where ρ was defined by $\rho =\gamma \overset{-}{q}$, $\overset{-}{q}=\frac{1}{Mp}\underset{j}{\sum }\underset{\mu }{\sum }q_{j\mu }$, as in the weight coding. Although normalization factor ρ slightly overestimates the connectivity, the resultant difference was negligibly small in our model setting. In the model with inhomogeneous input-activity variance, we instead used $\overset{-}{q}=\frac{1}{Mp}\underset{j}{\sum }\underset{\mu }{\sum }\theta_{j\mu }∕\sigma_{X}^{2}$ to suppress variability. For the cut-off coding strategy, the synaptic weight was chosen as *w*_*ij*_ = *w*_μ*j*_ = *q*_*jμ*_∕ρ_*o*_ where ρ_*o*_ is the mean connection probability. Based on these synaptic weights, for each output neuron, we selected *M*ρ_*o*_ largest synaptic connections, and eliminated all other connections. Thus, connection matrix *C* was given as $c_{ij}={\left[\underset{j^{\prime }}{\sum }{\left[w_{ij}\leq w_{ij^{\prime }}\right]}_{+}\leq M\rho_{o}\right]}_{+}$, where [true]_+_ = 1, [false]_+_ = 0. When multiple connections have the same weight, we randomly selected the connections so that the total number of inbound connections becomes *M*ρ_*o*_. Finally, in the random connection strategy, synaptic weights and connections were determined as *w*_*ij*_ = *w*_μ*j*_ = *q*_*jμ*_∕ρ_*o*_, Pr [*c*_*ij*_ = 1] = ρ_*o*_.

#### Synaptic weight learning

To perform maximum likelihood estimation from output neuron activity, synaptic weight matrix between input neurons and output neurons should provide a reverse model of input neuron activity. If the reverse model is faithful, KL-divergence between the true input and the estimated distributions $D_{KL}\left[p^{*}\left(r_{X}^{t}\right)||p\left(r_{X}^{t}|C,W\right)\right]$ would be minimized (Dayan et al., 1995; Nessler et al., 2013). Therefore, synaptic weights learning can be performed by ${\text{argmin}}_{W}D_{KL}\left[p^{*}\left(r_{X}^{t}\right)||p\left(r_{X}^{t}|C,W\right)\right]$. Likelihood $p\left(r_{X}^{t}|C,W\right)$ is approximated as

(10) $$\begin{array}{l} p(r_{X}^{t}|C,W)\propto \sum_{\mu }p(r_{X}^{t}|s^{t}=\mu ,C,W)p(s^{t}=\mu |C,W)\\ \;=\sum_{\mu }p(s^{t}=\mu |C,W)\exp\\ \;\left[\sum_{j}\left(h(\theta_{j,\mu }^{C,W})g(r_{X,j}^{t})-A(\theta_{j,\mu }^{C,W})+B(r_{X,j}^{t})\right)\right]\\ \;≃\sum_{\mu }p(s^{t}=\mu )\exp\\ \;\left[\sum_{j}\left(q_{j\mu }^{C,W}g(r_{X,j}^{t})-\alpha (q_{j\mu }^{C,W})+B(r_{X,j}^{t})\right)\right]\text{​}. \end{array}$$

$\theta_{j,\mu }^{C,W}$ in the second line is the average response estimated from connectivity matrix *C*, and weight matrix *W*. In the last equation, $q_{j\mu }^{C,W}$ is substituted for $h\left(\theta_{j,\mu }^{C,W}\right)$. If we approximate the estimated parameter $q_{j\mu }^{C,W}$ with $q_{j\mu }^{C,W}≃\rho_{o}w_{ij}$ by using the average connectivity ρ_*o*_, a synaptic weight plasticity rule is given by stochastic gradient descending as

(11) $$\begin{array}{l} \Delta w_{ij}\propto \frac{\partial \log p\left(r_{X}^{t}|C,W\right)}{\partial w_{ij}}\\ \;=p\left(s^{t}=\mu |r_{X}^{t},C,W\right)\rho_{o}\left(g\left(r_{X,j}^{t}\right)\;-\;\alpha^{\prime }\left(\rho_{o}w_{ij}\right)\right)\\ \;≃r_{Y,i}^{t}\rho_{o}\left(g\left(r_{X,j}^{t}\right)-\alpha^{\prime }\left(\rho_{o}w_{ij}\right)\right). \end{array}$$

Especially, in a Gaussian model, the synaptic weight converges to the weight coding as $w_{ij}=\langle r_{Y,i}^{t}r_{X,j}^{t}∕\left(\sigma_{X}^{2}\rho_{o}r_{Y,i}^{t}\right)\rangle =q_{j\mu }∕\rho_{o}$, where μ is the external state that output neuron *i* learned to represent (i.e., *i* ∈ Ω_μ_).

As we were considering population representation, in which the total number of output neuron is larger than the total number of external states (i.e., *p* < *N*), there is a redundancy in representation. Thus, to make use of most of population, homeostatic constraint is necessary. For homeostatic plasticity, we set a constraint on the output firing rate. By combining two terms, synaptic weight plasticity rule is given as

(12) $$\Delta w_{ij}=\frac{\eta_{X}}{\gamma }\left(r_{Y,i}^{t}\left[g(r_{X,j}^{t})-\alpha^{\prime }\left(\rho_{o}w_{ij}\right)\right]+b_{h}\left[r_{Y}^{o}/N-r_{Y,i}^{t}\right]\right).$$

By changing the strength of homeostatic plasticity *b*_*h*_, the network changes its behavior. The learning rate is divided by γ, because the mean of *w* is proportional to 1/γ. Although, this learning rule is unsupervised, each output neuron naturally selects an external state in self-organization manner.

#### Synaptic connection learning

Wiring plasticity of synaptic connection can be given in a similar manner. As shown in Figure 3, if the synaptic connection structure of network is correlated with the external model, the learning performance typically gets better. Therefore, by considering ${\text{argmin}}_{\rho }D_{KL}\left[p^{*}\left(r_{X}^{t}\right)||p\left(r_{X}^{t}|\rho ,W\right)\right]$, the update rule of connection probability is given as

(13) $$\Delta \rho_{ij}\propto r_{Y,i}^{t}w_{o}\left[g(r_{X,j}^{t})-\alpha^{\prime }(\rho_{ij}w_{o})\right].$$

Here, we approximated *w*_*ij*_ with its average value *w*_*o*_. In this implementation, if synaptic weight is also plastic, convergence of *D*_*KL*_ is no longer guaranteed, yet as shown in Figure 3, redundant representation robustly provides a good heuristic solution.

Let us next consider the implementation of the rewiring process with local spine elimination and creation based on the connection probability ρ_*ij*_. To keep the detailed balance of connection probability, creation probability *c*_*p*_(ρ) and elimination probability *e*_*p*_(ρ) need to satisfy

$$\left(1-\rho )c_{p}(\rho )=\rho e_{p}(\rho \right)$$

The simplest functions that satisfy above equation is *c*_*p*_(ρ) ≡ ρ*∕τ*_*c*_, *e*_*p*_(ρ) ≡ (1−ρ)∕τ_*c*_. In the simulation, we implemented this rule by changing *c*_*ij*_ from 1 to 0 with probability (1−ρ)∕τ_*c*_ for every connection with *c*_*ij*_ = 1, and shift *c*_*ij*_ from 0 to 1 with probability ρ*∕τ*_*c*_ for non-existing connection (*c*_*ij*_ = 0) at every time step.

#### Dual hebbian rule and estimated transfer entropy

The results in the main texts suggest that non-random synaptic connection structure can be beneficial either when that increases estimated transfer entropy or is correlated with the structure of the external model. To derive dual Hebbian rule, we used the latter property, yet in the simulation, estimated transfer entropy also increased by the dual Hebbian rule. Here, we consider relationship of two objective functions. Estimation of the external state from the sampled inputs is approximated as

(14) $$\begin{array}{l} \langle p(s^{t}=\mu )|\{c_{ij}r_{X,j}^{t}\}\rangle i\in \Omega_{\mu }≃\frac{1}{\left|\Omega_{\mu }\right|}\\ \sum_{i\in \Omega_{\mu }}\frac{\begin{array}{l} p(s^{t}=\mu )\exp(\underset{j}{\sum }\rho_{ij}[q_{\mu j}g(r_{X,j}^{t})-\alpha (q_{\mu j})+B(r_{X,j}^{t})]) \end{array}}{\begin{array}{l} \underset{\nu }{\sum }p(s^{t}=\nu )\exp(\underset{j}{\sum }c_{ij}[q_{\nu j}g(r_{X,j}^{t})-\alpha (q_{\nu j})+B(r_{X,j}^{t})]) \end{array}} \end{array}$$

Therefore, by considering stochastic gradient descending, an update rule of ρ_*ij*_ is given as

(15) $$\begin{array}{l} \Delta \rho_{ij}\propto \left(1+\log r_{Y,i}^{t}/r_{Y}^{o}\right)r_{Y,i}^{t}\\ \left[g(r_{X,j}^{t})-\alpha (q_{\mu j})/q_{\mu j}+B(r_{X,j}^{t})/q_{\mu j}\right] \end{array}$$

If we compare this equation with the equation for dual Hebbian rule (Equation 13), both of them are monotonically increasing function of $r_{Y,i}^{t}$ and have the same dependence on $g\left(r_{X,j}^{t}\right)$ although normalization terms are different. Thus, the change directions in dynamics given by Equation (13) and (15) have on average positive cross-correlation, hence under an adequate normalization, the inner product of change direction becomes positive on average. Therefore, although dual Hebbian learning rule does not maximize the estimated maximum transfer entropy, the rule rarely diminishes it.

#### Gaussian model

We constructed mean response probabilities ${\left\{\theta_{j\mu }\right\}}_{j=1,\;\ldots ,M}^{\mu =1,\;\ldots ,p}$ by following 2 steps. First, non-normalized response probabilities ${\left\{{\tilde{\theta }}_{j\mu }\right\}}_{j=1,\;\ldots ,M}^{\mu =1,\ldots ,p}$ were chosen from a truncated normal distribution *N*(μ_*M*_, σ_*M*_) defined on [0, ∞). Second, we defined ${\left\{\theta_{j\mu }\right\}}_{j=1,\ldots ,M}^{\mu =1,\ldots ,p}$ by $\theta_{j\mu }={\tilde{\theta }}_{j\mu }∕Z_{\mu }$, where $Z_{\mu }=r_{X}^{o}∕\sqrt{\overset{M}{\underset{j=1}{\sum }}{\tilde{\theta }}_{j\mu }∕M}$. Truncated normal distribution was chosen for performing Gaussian approximation in analytical calculation (see Section Evaluation of Performances in Weight Coding and Connectivity Coding). When the noise follows a Gaussian distribution, the response functions is given as

$$f\left(r_{X,j}^{t}|s^{t}=s_{\mu }\right)=\exp\left[-\frac{1}{2\sigma_{X}^{2}}{\left(r_{X,j}^{t}-\theta_{j\mu }\right)}^{2}-\log\left(\sqrt{2\pi }\sigma_{x}\right)\right],$$

thus functions in Equation (5) are uniquely defined as

(16) $$\begin{array}{l} h(\theta )=\frac{\theta }{\sigma_{x}^{2}},g(r)=r,A(\theta )=\frac{\theta^{2}}{2\sigma_{x}^{2}}+\log(\sqrt{2\pi }\sigma_{x}),\\ B(r)=-\frac{r^{2}}{2\sigma_{x}^{2}}. \end{array}$$

Because $h^{-1}\left(q\right)=\sigma_{x}^{2}q$, α(*q*) is given as $\alpha \left(q\right)\equiv A\left(h^{-1}\left(q\right)\right)\;=\;\sigma_{x}^{2}q^{2}∕2+\log\left(\sqrt{2\pi }\sigma_{x}\right)$. By substituting *g(r)* in Equation (8) with *g(r)* in Equation (16), the neural dynamics is given as

(17) $$r_{Y,i}^{t}=r_{Y}^{o}\exp\left[\sum_{j\;=\;1}^{M}c_{ij}\left(w_{ij}r_{X,j}^{t}-w_{o}\right)-I_{inh}^{\;t}\right].$$

Thus, in this model setting, optimal inference in all-to-all connection is given by $w_{ij}=q_{j\mu }∕\sigma_{X}^{2}$. Similarly, dual Hebbian rule becomes

(18) $$\Delta w_{ij}=\frac{\eta_{X}}{\gamma }\left(r_{Y,i}^{t}\left[r_{X,j}^{t}-\sigma_{X}^{2}\rho_{o}w_{ij}\right]+b_{h}\left[r_{Y}^{o}/N-r_{Y,i}^{t}\right]\right)$$

(19) $$\Delta \rho_{ij}=\eta_{\rho }r_{Y,i}^{t}\left(r_{X,j}^{t}-\sigma_{x}^{2}\rho_{ij}w_{o}\right).$$

#### Poisson model

For Poisson model, we defined mean response probabilities ${\left\{\theta_{j\mu }\right\}}_{j=1,\ldots ,M}^{\mu =1,\ldots ,p}$ from a log-normal distribution instead of a normal distribution. Non-normalized values were sampled from a truncated log-normal distribution $\log N\left(\mu_{M}^{p},\sigma_{M}^{p}\right)$ defined on $\left(l_{min}^{p},l_{max}^{p}\right)$. Normalization was performed as $\theta_{j\mu }={\tilde{\theta }}_{j\mu }∕Z_{\mu }$ for ${\left\{{\tilde{\theta }}_{j\mu }\right\}}_{j=1,\ldots ,M}^{\mu =1,\ldots ,p}$, where $Z_{\mu }=r_{X}^{o}M∕\underset{j}{\sum }\theta_{j\mu }$. Because the noise follows a Poisson distribution *p*(*r*|θ) = exp[−*q* + *r* log *q* − log *r*!], the response functions are given as

(20) h(θ)=logθ,g(r)=r,A(θ)=θ,B(r)=−logr!.

As a result, α(*q*) is defined as α(*q*) ≡ *A*(*h*^−1^(*q*)) = *e*^*q*^. By substituting them to the original equations, the neural dynamics also follows Equation (17). If connection is all-to-all, by setting *w*_*ij*_ = log(θ_*jμ*_∕θ_*o*_) for *i* ∈ Ω_μ_, optimal inference is achievable. Here, we normalized θ_*j*_ by θ_*o*_, which is defined as $\theta_{o}=\frac{1}{2}\underset{j,\mu }{\min}\theta_{\mu j}$, in order to keep synaptic weights in positive values. Note that, theoretically speaking, θ_*o*_ can be any value satisfying $0<\theta_{o}<\underset{j,\mu }{\min}\theta_{j\mu }$, yet here we used $\theta_{o}=\frac{1}{2}\underset{j,\mu }{\min}\theta_{\mu j}$ for numerical stability.

Learning rules for synaptic weight and connection are given as

(21) $$\begin{array}{l} \Delta w_{ij}=\frac{\eta_{x}}{\gamma }(r_{Y,i}^{t}\left[r_{X,j}^{t}-\theta_{min}\exp[\rho_{o}w_{ij}]\right]\;+\\ \;b_{h}\left[r_{Y}^{o}/N-r_{Y,i}^{t}\right]) \end{array}$$

(22) $$\Delta \rho_{ij}=\eta_{\rho }r_{Y,i}^{t}\left(r_{X,j}^{t}-\theta_{min}\exp(\rho_{ij}w_{o})\right).$$

Note that the first term of the synaptic weight learning rule coincides with a previously proposed optimal learning rule for spiking neurons (Habenschuss et al., 2013; Nessler et al., 2013). In calculation of model error, error was calculated as $d=\sqrt{\frac{1}{pM}\sum_{\mu }\sum_{j}{\left({\tilde{q}}_{j\mu }-q_{\;j\mu }^{*}\right)}^{2}}$, where estimated parameter $\left\{{\tilde{q}}_{j\mu }\right\}$ was given by ${\tilde{q}}_{j\mu }=\frac{\langle q_{j\mu }^{*}\rangle {\overset{-}{q}}_{j\mu }}{\sum_{q}\sum_{j}{\overset{-}{q}}_{j\mu }∕pM}$. Here, $\langle q_{j\mu }^{*}\rangle$ represents the mean of true {*q*_*jμ*_}, and non-normalized estimator ${\overset{-}{q}}_{j\mu }$ was calculated as ${\overset{-}{q}}_{j\mu }=\frac{1}{\langle c_{ij}\rangle |\Omega_{\mu }|\;}\sum_{i\in \Omega_{\mu }}c_{ij}w_{ij}$. In Supplementary Figure 1D, estimation from connectivity was calculated from ${\overset{-}{q}}_{j\mu }^{C}=\frac{1}{\langle c_{ij}\rangle |\Omega_{\mu }|\;}\sum_{i\in \Omega_{\mu }}c_{ij}$, and similarly, estimation from weights was calculated by ${\overset{-}{q}}_{j\mu }^{W}=\frac{1}{|\Omega_{\mu }|\underset{i\in \Omega_{\mu }}{\sum }c_{ij}}\underset{i\in \Omega_{\mu }}{\sum }c_{ij}w_{ij}$. For parameters, we used $\mu_{M}^{p}=0.0$, $\sigma_{M}^{p}=1.0$, $l_{min}^{p}=0.2$, $l_{max}^{p}=20.0$, *w*_*o*_ = 1∕γ, $r_{X}^{o}=0.3$, and for other parameters, we used same values with the Gaussian model.

### Analytical evaluations

#### Evaluation of performances in weight coding and connectivity coding

In Gaussian model, we can analytically evaluate the performance in two coding schemes. As the dynamics of output neurons follows $r_{Y,i}=r_{Y}^{o}\exp\left[\sum_{j}c_{ij}\left(w_{ij}r_{X,j}^{t}-w_{o}\right)-I_{inh}^{t}\right],$ membrane potential variable *u*_*i*_, which is defined as

(23) $$u_{i}\equiv \sum_{j}c_{ij}(w_{ij}r_{X,j}^{t}-w_{o}),$$

determines firing rates of each neuron. Because {θ_*jμ*_} is normalized with $\sum_{j=1}^{M}\theta_{j\mu }^{2}∕M={\left(r_{X}^{o}\right)}^{2}$, mean and variance of {θ_*jμ*_} are given as

(24) $$\mu_{\theta }=\frac{\mu_{M}r_{X}^{o}}{\sqrt{\mu_{M}^{2}+\sigma_{x}^{2}}},\sigma_{\theta }^{2}=\frac{{\left(\sigma_{M}r_{X}^{o}\right)}^{2}}{\mu_{M}^{2}+\sigma_{M}^{2}},$$

where μ_*M*_ and σ_*M*_ are the mean and variance of the original non-normalized truncated Gaussian distribution $\left\{{\tilde{\theta }}_{j\mu }\right\}$. Because both *r*_*X, j*_ and {θ_*jμ*_} approximately follow Gaussian distribution, *u*_*i*_ is expected to follow Gaussian. Therefore, by evaluating its mean and variance, we can characterize the distribution of *u*_*i*_ for a given external state (Babadi and Sompolinsky, 2014).

Let us first consider the distribution of *u*_*i*_ in the weight coding. In weight coding scheme, *w*_*ij*_ and *c*_*ij*_ are defined as

(25) $$w_{ij}=\theta_{j\mu }/\rho \sigma_{x}^{2},\mathrm{Pr}\left[c_{ij}=1\right]=\rho$$

where $\rho =\gamma \mu_{\theta }∕\sigma_{x}^{2}$. By setting $w_{o}=\mu_{\theta }^{2}∕\left(\rho \sigma_{X}^{2}\right)$, the mean membrane potential of output neuron *i* selective for given signal (i.e., *i* ∈ Ω_μ_ for *s*^*t*^ = μ) is calculated as,

$$\langle u_{i}\rangle =\langle \sum_{j}\left(\theta_{j\mu }^{2}-{\langle \theta_{j\mu }\rangle }^{2}\right)/\sigma_{x}^{2}\rangle =M\sigma_{\theta }^{2}/\sigma_{x}^{2}.$$

Similarly, the variance of *u*_*i*_ is given as

(26) $$\begin{array}{l} \langle {\left(u_{i}-\langle u_{i}\rangle \right)}^{2}\rangle =\\ \langle (\frac{1}{\rho \sigma_{X}}\underset{j}{\sum^{\text{​}}}c_{ij}\theta_{j\mu }\zeta_{j}+\frac{1}{\rho \sigma_{X}^{2}}\underset{j}{\sum^{\text{​}}}(c_{ij}-\rho )(\theta_{j\mu }^{2}-\mu_{\theta }^{2})+\\ \frac{1}{\sigma_{X}^{2}}\underset{j}{\sum^{\text{​}}}(\theta_{j\mu }^{2}-[\mu_{\theta }^{2}+\sigma_{\theta }^{2}]){)}^{2}\rangle \\ =\frac{M}{\rho \sigma_{X}^{2}}(\mu_{\theta }^{2}+\sigma_{\theta }^{2})+\frac{M\sigma_{\theta }^{2}}{\rho \sigma_{X}^{4}}[2(2\mu_{\theta }^{2}+\sigma_{\theta }^{2})+(1-\rho )\sigma_{\theta }^{2}] \end{array}$$

where ζ_*j*_ is a Gaussian random variable. On the other hand, if output neuron *i* is not selective for the presented stimuli (if *s*^*t*^≠μ and *i* ∈ Ω_μ_), *w*_*ij*_ and *r*_*X, j*_ are independent. Thus, the mean and the variance of *u*_*i*_ are given as,

$$\langle u_{i}\rangle =0,\;\langle {\left(u_{i}-\langle u_{i}\rangle \right)}^{2}\rangle =\frac{M}{\rho \sigma_{x}^{2}}(\mu_{\theta }^{2}+\sigma_{\theta }^{2})+\frac{M\sigma_{\theta }^{2}}{\rho \sigma_{x}^{4}}\left(2\mu_{\theta }^{2}+\sigma_{\theta }^{2}\right)$$

In addition to that, due to feedforward connection, output neurons show noise correlation. For two output neurons *i* and *l* selective for different states (i.e., *i* ∈ Ω_μ_ and *l*∉Ω_μ_), the covariance between *u*_*i*_ and *u*_*l*_ satisfies

$$\langle \left(u_{i}-\langle u_{i}\rangle )(u_{l}-\langle u_{l}\rangle \right)\rangle =\langle \rho^{2}\sum_{j}w_{ij}w_{lj}{\left(r_{X,j}-\theta_{j\mu }\right)}^{2}\rangle =M\mu_{\theta }^{2}/\sigma_{x}^{2}$$

Therefore, approximately (*u*_*i*_, *u*_*l*_) follows a multivariable Gaussian distributions

(27) $$\begin{array}{l} \left(\begin{array}{c} u_{i}\\ u_{l} \end{array}\right)=N(\left(\begin{array}{c} \frac{M\sigma_{\theta }^{2}}{\sigma_{x}^{2}}\\ 0 \end{array}\right),(\frac{M\left(\mu_{\theta }^{2}\;+\;\sigma_{\theta }^{2}\right)}{\rho \sigma_{X}^{2}}\\ \begin{array}{cc} +\frac{M\sigma_{\theta }^{2}\left[2\left(2\mu_{\theta }^{2}\;+\;\sigma_{\theta }^{2}\right)\;+\;\left(1-\rho \right)\sigma_{\theta }^{2}\right]}{\rho \sigma_{X}^{4}} & \frac{M\mu_{\theta }^{2}}{\sigma_{x}^{2}}\\ \frac{M\mu_{\theta }^{2}}{\sigma_{x}^{2}} & \frac{M(\mu_{\theta }^{2}\;+\;\sigma_{\theta }^{2})}{\rho \sigma_{x}^{2}}+\frac{M\sigma_{\theta }^{2}\left(2\mu_{\theta }^{2}\;+\;\sigma_{\theta }^{2}\right)}{\rho \sigma_{x}^{4}} \end{array}))\text{​​}. \end{array}$$

In maximum likelihood estimation, the estimation fails if a non-selective output neuron shows higher firing rate than the selective neuron. When there are two output neurons, probability for such an event is calculated as

ϵw=Pr[∑jclj(wljrX,jt−wo)>∑jcij(wijrX,jt−wo)|st     =μ,i∈Ωμ,l∈Ωμ].

In the simulation, there are *p* − *1* distractors per one selective output neuron. Thus, approximately, accuracy of estimation was evaluated by ${\left(1-\epsilon_{w}\right)}^{p-1}$. In Figure 2B, we numerically calculated this value for the analytical estimation.

Similarly, in connectivity coding, *w*_*ij*_ and *c*_*ij*_ are given as

$$w_{ij}=1/\gamma ,\mathrm{Pr}[c_{ij}=1]=\rho_{ij},\;\rho_{ij}=\gamma \theta_{j\mu }/\sigma_{x}^{2}.$$

By setting *w*_*o*_ = μ_θ_∕γ, from a similar calculation done above, the mean and the variance of (*u*_*i*_, *u*_*l*_) are derived as

(28) $$\begin{array}{l} \left(\begin{array}{c} u_{i}\\ u_{l} \end{array}\right)=N(\left(\begin{array}{c} \frac{M\sigma_{\theta }^{2}}{\sigma_{x}^{2}}\\ 0 \end{array}\right),\\ \;\left(\begin{array}{cc} \frac{M\mu_{\theta }}{\gamma }+\frac{M\sigma_{\theta }^{2}\left[\mu_{\theta }\sigma_{x}^{2}-\gamma \sigma_{\theta }^{2}\right]}{\gamma \sigma_{x}^{4}} & \frac{M\mu_{\theta }^{2}}{\sigma_{x}^{2}}+\frac{M\mu_{\theta }^{2}\sigma_{\theta }^{2}}{\sigma_{x}^{4}}\\ \frac{M\mu_{\theta }^{2}}{\sigma_{x}^{2}}+\frac{M\mu_{\theta }^{2}\sigma_{\theta }^{2}}{\sigma_{x}^{4}} & \frac{M\mu_{\theta }}{\gamma }+\frac{M\mu_{\theta }\sigma_{\theta }^{2}}{\gamma \sigma_{x}^{2}} \end{array}\right)) \end{array}$$

If we compare the two coding schemes, means are the same for two coding schemes, and as γ satisfies $\gamma =\sigma_{x}^{2}\rho \mu_{\theta }$, variance of non-selective output neuron are similar. The main difference is the second term of signal variance. In the weight coding, signal variance is proportional to *1/*γ, on the other hand, in the connectivity coding, the second term of signal variance is negative, and does not depend on the connectivity. As a result, in the adequately sparse regime, firing rate variability of selective output neuron becomes smaller in connectivity coding, and the estimation accuracy is better. In the sparse limit, the first term of variance becomes dominant and both schemes do not work well, consequently, the advantage for connectivity coding disappears. Coefficient of variation calculated for signal terms is indeed smaller in connectivity coding scheme (blue and red lines in Figure 2C), and the same tendency is observed in simulation (cyan and orange lines in Figure 2C).

#### Optimality of connectivity

To evaluate optimality of a given connection matrix *C*, we calculated the posterior probability of the external states estimated from *C* and *r*_*X*_, and compared then to that from the fully connected network *C*_*all*_. Below, we denote the mean KL-divergence ${\langle D_{KL}\left[p\left(s^{t}|r_{X},C_{all}\right)||p\left(s^{t}|r_{X},C\right)\right]\rangle }_{r_{X}}$ as *I(C*_*all*_*,C)* for readability. When the true external state is *s*^*t*^ = ν, firing rates of input neurons are given by $r_{X,j}^{t}$ ~ *N*(θ_*jν*_, σ_*X*_), hence this *I(C*_*all*_*,C)* is approximately evaluated as

$$\begin{array}{l} I\left(C_{all},C\right)\approx \frac{1}{p}\sum_{\nu }{\langle D_{KL}\left[p\left(s^{t}|r_{X|\nu },C_{all}\right)||p\left(s^{t}|r_{X|\nu },C\right)\right]\rangle }_{r_{X}}\\ \;\approx \frac{1}{p}\sum_{\nu }D_{KL}[{\langle p\left(s^{t}|\left\{\theta_{j\nu }+\sigma_{X}\zeta_{j}\right\},C_{all}\right)\rangle }_{\left\{\zeta_{j}\right\}}\\ \;||{\langle p\left(s^{t}|\left\{\theta_{j\nu }+\sigma_{X}\zeta_{j}\right\},C\right)\rangle }_{\left\{\zeta_{j}\right\}}] \end{array}$$

where {ζ_*j*_} are Gaussian random variables, and *C*_*all*_ represents the all-to-all connection matrix. By taking integral over Gaussian variables, the posterior probability is evaluated as

$$\begin{array}{l} {\langle p\left(s^{t}=\mu |\left\{\theta_{j\nu }+\sigma_{X}\zeta_{j}\right\},C\right)\rangle }_{\left\{\zeta_{j}\right\}}\\ ≅\frac{1}{\left|\Omega_{\mu }\right|}\sum_{i\in \Omega_{\mu }}\frac{\exp\left(\phi_{\mu \nu }^{i,C}+\frac{1}{2}\psi_{\mu }^{i,C}\right)}{\sum_{\mu^{\prime }}\exp\left(\phi_{\mu^{\prime }\nu }^{i,C}+\frac{1}{2}\psi_{\mu^{\prime }}^{i,C}\right)}\equiv p_{\nu }\left(s^{t}=\mu |C\right), \end{array}$$

where

$$\phi_{\mu \nu }^{i,C}\equiv \sum_{j}c_{ij}\;\left(2\theta_{\mu j}\theta_{\nu j}-\theta_{\mu j}^{2}\right)/\left(2\sigma_{X}^{2}\right),\;\psi_{\mu }^{i,C}\equiv \sum_{j}c_{ij}{\left(\theta_{\mu j}/\sigma_{X}\right)}^{2}.$$

Thus, the KL-divergence between estimations by two connection structures *C*_*all*_ and *C* is approximated as:

(29) $$I\left(C_{all},C\right)\approx \frac{1}{p}\sum_{\nu }\sum_{\mu }p_{\nu }\left(s^{t}=\mu |C_{all}\right)\log\frac{p_{\nu }\left(s^{t}=\mu |C_{all}\right)}{p_{\nu }\left(s^{t}=\mu |C\right)}$$

In the black lines in Figures 3C–E, we maximized the approximated KL-divergence *I(C*_*all*_*, C)* with a hill-climbing method from various initial conditions, thus the lines may not be the exact optimal, but rather lower bounds of the optimal performance. Information gain by a connection *c*_*ij*_ was evaluated by

(30) $$\Delta I_{ij}\equiv {\langle I\left(C_{\;all},C\right)-I\left(C_{all},C+\eta_{ij}\right)\rangle }_{C},$$

where η_*ij*_ is a *N*×*M* matrix in which only (*i, j*) element takes 1, and all other elements are 0. In Figure 3B, we took average over 1000 random connection structures with connection probability ρ = 0.1.

### Model settings

#### Details of simulation

In the simulation, the external variable *s*^*t*^ was chosen from 10 discrete variables (*p* = 10) with equal probability (Pr [*s*^*t*^ = *q*] = 1/*p*, for all *q*). The mean response probability θ_*jμ*_ was given first by randomly chosen parameters ${\left\{{\tilde{\theta }}_{j\mu }\right\}}_{j=1,\ldots ,M}^{\mu =0,\ldots ,p-1}$ from the truncated normal distribution *N*(μ_*M*_, σ_*M*_) in [0, ∞), and then normalized using $\theta_{j\mu }={\tilde{\theta }}_{j\mu }∕Z_{\mu }$, where $Z_{\mu }=r_{X}^{o}∕\sqrt{\overset{M}{\underset{j=1}{\sum }}{\tilde{\theta }}_{j\mu }∕M}$. Mean weight *w*_*o*_ was defined as $w_{o}=r_{X}^{o}∕\gamma$. The normalization factor *h*_*w*_ was defined as $h_{w}=\overset{-}{q}∕\gamma$ in Figures 1, 2, 4, 5, where $\overset{-}{q}=\frac{1}{Mp}\underset{j}{\sum }\underset{\mu }{\sum }\theta_{j\mu }∕\sigma_{X}^{2}$, and as $h_{w}=r_{X}^{o}∕\gamma$ in Figures 6, 7, as the mean of θ depends on κ_*m*_. In Figure 3, we used $h_{w}=\overset{-}{q}∕\gamma$ for the dual coding, and $h_{w}=\overset{-}{q}∕\rho_{o}$ for the rest. Average connectivity $\overset{-}{\rho }$ was calculated from the initial connection matrix of each simulation. In the calculation of the dynamics, for the membrane parameter $v_{i}\equiv \underset{j}{\sum }c_{ij}\left(w_{ij}r_{X,j}^{t}-h_{w}\right)$, a boundary condition $v_{i}>\underset{\ell }{\max}\left\{v_{\ell }-v_{d}\right\}$ was introduced for numerical convenience, where *v*_*d*_ = −60 and ℓ is index for the output neurons. In addition, synaptic weight *w*_*ij*_ was bounded to a non-negative value (*w*_*ij*_ > 0), and the connection probability was defined as ρ ∈ [0, 1]. For the dynamics of synaptic weight *w*_*ij*_ in Figures 4–8, we used Equation (2), and for the dynamics of connection probability ρ_*ij*_, we used Equation (3) in Figures 5, 6, and Equation (4) in Figures 6I, 7, 8, unless stated otherwise. In the rest of figures, both *w*_*ij*_ and ρ_*ij*_ were kept constants on the values described in Sections Weight Coding and Connectivity Coding and Dual Coding and Cut-Off Coding. For simulations with synaptic weight learning, initial weights were defined as $w_{ij}=\left(1+\sigma_{w}^{init}\zeta \right)∕\gamma$, where $\sigma_{w}^{init}$ = 0.1, and ζ is a Gaussian random variable. Similarly, in the simulation with structural plasticity, the initial condition for the synaptic connection matrix was defined as $\mathrm{Pr}\left[c_{ij}=1\right]=\gamma \langle \theta_{j\mu }\rangle ∕\sigma_{x}^{2}$. In both the dual Hebbian rule and the approximated dual Hebbian rule, the synaptic weight of a newly created spine was given as $w_{ij}=\left(1+\sigma_{w}^{init}\zeta \right)w_{o}$, for a random Gaussian variable ζ ← *N*(0, 1). In Figure 8, simulations were initiated at −20 days (i.e., 2 × 10^6^ steps before stimulus onset) to ensure convergence for the control condition. For model parameters, μ_*M*_ = 1.0, σ_*M*_ = 1.0, σ_*X*_ = 1.0, *M* = 200, *N* = 100 $r_{X}^{{}^{\circ}}=$ 1.0, and $r_{Y}^{{}^{\circ}}=$ 1.0 were used, and for learning-related parameters, η_*X*_ = 0.01, *b*_*h*_ = 0.1, η_ρ_= 0.001, τ_*c*_ = 10^6^, *T*_2_ = 10^5^, and κ_*m*_ = 0.5 were used. In Figures 7, 8, η_ρ_ = 0.0001, $\tau_{c}=3\times 10^{5}$, and γ = 0.6 were used, unless otherwise stated.

#### Accuracy of estimation

The accuracy was measured with the bootstrap method. By using data from *t*-*T*_*o*_ < = *t'* < *t*, the selectivity of output neurons was first decided. Ω_μ_ was defined as a set of output neurons that represents external state μ. Neuron *i* belongs to set Ω_μ_ if *i* satisfies

$$\mu =\underset{\mu^{\prime }}{\arg\max}\frac{\sum_{t^{\prime }\;=\;t-T_{o}}^{t}{\left[s^{t}=\mu^{\prime }\right]}_{+}r_{Y,i}^{t}}{\sum_{t^{\prime }\;=\;t-T_{o}}^{t}{\left[s^{t}=\mu^{\prime }\right]}_{+}},$$

where operator [X]_+_ returns 1 if X is true; otherwise, it returns 0. By using this selectivity, based on data from *t* < = *t'* < *t*+*T*_*o*_, the accuracy was estimated as

$$\frac{1}{T_{o}}\sum_{t^{\prime }\;=\;t}^{t+T_{o}-1}{\left[\frac{1}{\left|\Omega_{s^{t^{\prime }}}\right|}\sum_{i\in \Omega_{s^{t^{\prime }}}}r_{Y,i}^{t^{\prime }}>\underset{\mu \neq s^{t^{\prime }}}{\max}\frac{1}{\left|\Omega_{\mu }\right|}\sum_{i\in \Omega_{\mu }}r_{Y,i}^{t^{\prime }}\right]}_{tof}.$$

In this method, even if the connection structure between input and output layers is completely random, still the accuracy of estimation typically becomes slightly higher than the chance level (= 1.0/*p*) if output activity is not completely uniform, but the effect is almost negligible for the given model settings. In the simulation, *T*_*o*_ = 10^3^ was used because this value is sufficiently slow compared with weight change but sufficiently long to suppress variability.

#### Model error

Using the same procedure, model error was estimated as

$$d=\sqrt{\frac{1}{pM}\sum_{\mu \;=\;1}^{p}\sum_{j\;=\;1}^{M}{\left({\tilde{\theta }}_{j\mu }-\theta_{j\mu }\right)}^{2}},$$

where ${\tilde{\theta }}_{j\mu }$ represents the estimated parameter. ${\tilde{\theta }}_{j\mu }$ was estimated by

$${\bar{\theta }}_{j\mu }=\frac{1}{\langle c_{ij}\rangle \left|\Omega_{\mu }\right|}\sum_{i\in \Omega_{\mu }}c_{ij}w_{ij},{\tilde{\theta }}_{j\mu }=r_{o}^{X}{\bar{\theta }}_{j\mu }/r_{o}^{X}{\bar{\theta }}_{j\mu }\sqrt{\frac{1}{M}\sum_{j\;=\;1}^{M}{\bar{\theta }}_{j\mu }^{2}}.$$

In Figure 6E, the estimation of the internal model from connectivity was calculated by

$${\bar{\theta }}_{j\mu }^{C}=\frac{1}{\langle c_{ij}\rangle \left|\Omega_{\mu }\right|}\sum_{i\in \Omega_{\mu }}c_{ij}.$$

Similarly, the estimation from the synaptic weight in Figure 6F was performed with

$${\bar{\theta }}_{j\mu }^{W}=\frac{1}{\left|\Omega_{\mu }\right|}\sum_{i\in \Omega_{\mu }}c_{ij}w_{ij}/\sum_{i\in \Omega_{\mu }}c_{ij}.$$

#### Transfer entropy

Entropy reduction caused by partial information on input firing rates was evaluated by transfer entropy:

$$T_{E}={\langle H\left(s^{t}\right)-H\left(s^{t}|r_{X}^{t},C\right)\rangle }_{t},$$

where

$$\begin{array}{l} H\left(s^{t}|r_{X}^{t},C\right)=-\sum_{\mu \;=\;1}^{p}p\left(s^{t}=s_{\mu }|r_{X}^{t},C\right)\log p\left(s^{t}=s_{\mu }|r_{X}^{t},C\right)\\ \;≅-\sum_{\mu \;=\;1}^{p}{\langle p\left(s^{t}=s_{\mu }|\left\{c_{ij}r_{X,j}^{t}\right\}\right)\rangle }_{i\in \Omega_{\mu }}\\ \;\log{\langle p\left(s^{t}=s_{\mu }|\left\{c_{ij}r_{X,j}^{t}\right\}\right)\rangle }_{i\in \Omega_{\mu }}, \end{array}$$

$$\begin{array}{l} {\langle p\left(s^{t}=s_{\mu }|\left\{c_{ij}r_{X,j}^{t}\right\}\right)\rangle }_{i\in \Omega_{\mu }}≅\frac{1}{\left|\Omega_{\mu }\right|}\sum_{i\in \Omega_{\mu }}p\left(s^{t}=s_{\mu }\right)\\ \prod_{c_{ij}=1}p\left(r_{X,j}^{t}|s^{t}=s_{\mu }\right)=\frac{1}{\left|\Omega_{\mu }\right|}\sum_{i\in \Omega_{\mu }}\\ \frac{\begin{array}{l} p\left(s^{t}=s_{\mu }\right)\exp\left(\sum_{j=1}^{M}c_{ij}\left[q_{\mu j}g\left(r_{X,j}^{t}\right)-\alpha \left(q_{\mu j}\right)+B\left(r_{X,j}^{t}\right)\right]\right) \end{array}}{\begin{array}{l} \sum_{\nu }p\left(s^{t}=s_{\nu }\right)\exp\left(\sum_{j\;=\;1}^{M}c_{ij}\left[q_{\nu j}g\left(r_{X,j}^{t}\right)-\alpha \left(q_{\nu j}\right)+B\left(r_{X,j}^{t}\right)\right]\right) \end{array}}. \end{array}$$

Output group Ω_μ_ was determined as described above. Here, the true model was used instead of the estimated model to evaluate the maximum transfer entropy achieved by the network.

#### Spine dynamics

In Figure 8H, x-axes were calculated as follows:

$$\begin{array}{l} \begin{array}{c} \text{new spine formed during first}\\ \text{two days and persisted at day 7} \end{array}\\ =\langle \sum_{i,j}c_{ij}\left(7d\right)\left[1-c_{ij}\left(0d\right)\right]c_{ij}\left(2d\right)/\sum_{i,j}c_{ij}\left(7d\right)\rangle \\ \text{total new spine at day 7}\\ =\langle \sum_{i,j}c_{ij}\left(7d\right)\left[1-c_{ij}\left(0d\right)\right]/\sum_{i,j}c_{ij}\left(7d\right)\rangle \\ \text{Elimination of existing spines}\\ =\langle \sum_{i,j}c_{ij}\left(0d\right)\left[1-c_{ij}\left(7d\right)\right]/\sum_{i,j}c_{ij}\left(0d\right)\rangle \end{array}$$

Here, we denoted the connectivity at day *n* as *c*_*ij*_(*n*−d). For instance, “total new spine at day 7” is the mean ratio of spines exist at day 7 which was absent at day 0, to spine exist at day 7. In this method, transient processes, such as elimination and recreation during day 0–7, are dismissed, but such rapid rewiring is rare in the model, and experimental observations tend to be suffered from the same problem. Hence, we used simplified calculation as described above.

## Code availability

C++ codes of the simulation program is available at ModelDB (http://modeldb.yale.edu/181913).

## Author contributions

Conceived and designed the experiments: NH, TF. Performed the experiments: NH. Analyzed the data: NH. Wrote the paper: NH, TF.

### Conflict of interest statement

The authors declare that the research was conducted in the absence of any commercial or financial relationships that could be construed as a potential conflict of interest.

## Supplementary material

Supplementary Figure 1 **Results in Poisson model. (A)** An example of output neuron activity before (top) and after (bottom) synaptic weight learning at connectivity ρ = 0.25. **(B)** Synaptic weight matrices before (left) and after (right) learning. Both X-neurons and Y-neurons were sorted based on their preferred external states. **(C)** Accuracy of estimation at various timescale of rewiring τ_*c*_. **(D)** Model error calculated from connectivity (left) and synaptic weights (right). **(E)** Comparison of performance among the model without wiring plasticity (cyan), and dual Hebbian model(orange). Corresponding results in the Gaussian model are described in Figures 4A, 5F,G, 6E,F,I, respectively.
