# Supplementary figures and images for: Hebbian Wiring Plasticity Generates Efficient Network Structures for Robust Inference with Synaptic Weight Plasticity
Source: Front Neural Circuits. 2016 May 31;10:41. doi: 10.3389/fncir.2016.00041 (PMC4885844; doi:10.3389/fncir.2016.00041)

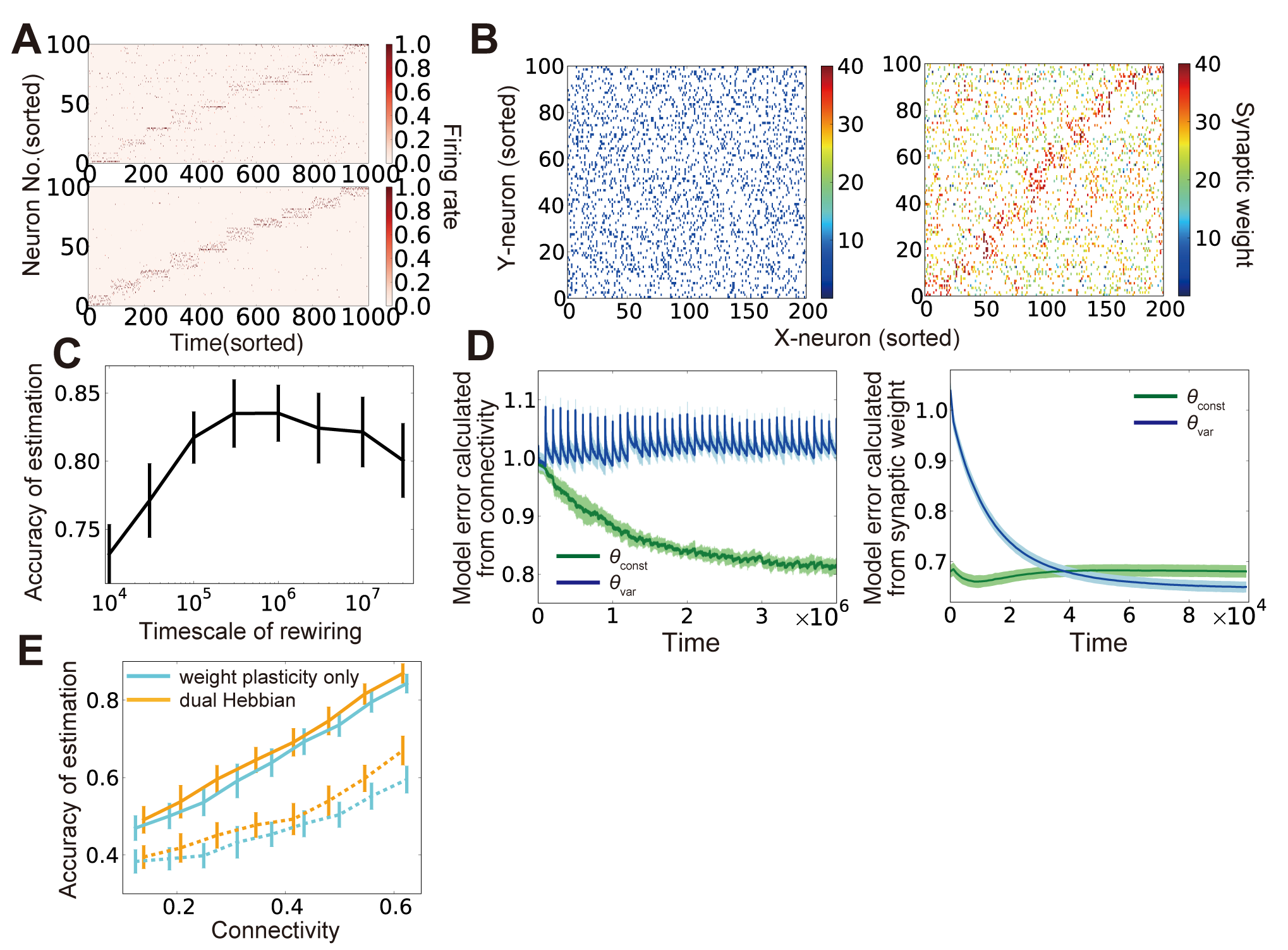

Supplement: Supplementary Figure 1 — Results in Poisson model. (A) An example of output neuron activity before (top) and after (bottom) synaptic weight learning at connectivity ρ = 0.25. (B) Synaptic weight matrices before (left) and after (right) learning. Both X-neurons and Y-neurons were sorted based on their preferred external states. (C) Accuracy of estimation at various timescale of rewiring τc. (D) Model error calculated from connectivity (left) and synaptic weights (right). (E) Comparison of performance among the model without wiring plasticity (cyan), and dual Hebbian model(orange). Corresponding results in the Gaussian model are described in Figures 4A, 5F,G, 6E,F,I, respectively. [file Image1.TIF]
